# Multimodal Analysis of Secondary Cerebellar Alterations After Pediatric Traumatic Brain Injury

**DOI:** 10.1001/jamanetworkopen.2023.43410

**Published:** 2023-11-15

**Authors:** Finian Keleher, Hannah M. Lindsey, Rebecca Kerestes, Houshang Amiri, Robert F. Asarnow, Talin Babikian, Brenda Bartnik-Olson, Erin D. Bigler, Karen Caeyenberghs, Carrie Esopenko, Linda Ewing-Cobbs, Christopher C. Giza, Naomi J. Goodrich-Hunsaker, Cooper B. Hodges, Kristen R. Hoskinson, Andrei Irimia, Marsh Königs, Jeffrey E. Max, Mary R. Newsome, Alexander Olsen, Nicholas P. Ryan, Adam T. Schmidt, Dan J. Stein, Stacy J. Suskauer, Ashley L. Ware, Anne L. Wheeler, Brandon A. Zielinski, Paul M. Thompson, Ian H. Harding, David F. Tate, Elisabeth A. Wilde, Emily L. Dennis

**Affiliations:** 1 TBI and Concussion Center, Department of Neurology, University of Utah School of Medicine, Salt Lake City; 2 George E. Wahlen Veterans Affairs Medical Center, Salt Lake City, Utah; 3 Department of Neuroscience, Central Clinical School, Monash University, Melbourne, Victoria, Australia; 4 Institute of Neuropharmacology, Neuroscience Research Center, Kerman University of Medical Sciences, Kerman, Iran; 5 Semel Institute for Neuroscience and Human Behavior, Department of Psychiatry and Biobehavioral Sciences, University of California, Los Angeles; 6 Brain Research Institute, University of California, Los Angeles; 7 Department of Psychology, University of California, Los Angeles; 8 Steve Tisch BrainSPORT Program, University of California, Los Angeles; 9 Department of Radiology, Loma Linda University Medical Center, Loma Linda, California; 10 Department of Psychology, Brigham Young University, Provo, Utah; 11 Neuroscience Center, Brigham Young University, Provo, Utah; 12 Cognitive Neuroscience Unit, School of Psychology, Deakin University, Geelong, Victoria, Australia; 13 Department of Rehabilitation and Human Performance, Icahn School of Medicine at Mount Sinai, New York, New York; 14 Children’s Learning Institute, Department of Pediatrics, University of Texas Health Science Center at Houston; 15 Division of Neurology, Department of Pediatrics, Mattel Children’s Hospital University of California, Los Angeles; 16 Department of Neurosurgery, David Geffen School of Medicine at the University of California, Los Angeles; 17 School of Social and Behavioral Sciences, Andrews University, Berrien Springs, Michigan; 18 Center for Biobehavioral Health, The Abigail Wexner Research Institute at Nationwide Children’s Hospital, Columbus, Ohio; 19 Department of Pediatrics, The Ohio State University College of Medicine, Columbus; 20 Ethel Percy Andrus Gerontology Center, Leonard Davis School of Gerontology, University of Southern California, Los Angeles,; 21 Department of Biomedical Engineering, Viterbi School of Engineering, University of Southern California, Los Angeles; 22 Emma Neuroscience Group, Emma Children’s Hospital, Amsterdam University Medical Centers, University of Amsterdam, Amsterdam, the Netherlands; 23 Department of Psychiatry, University of California, San Diego, La Jolla; 24 Department of Psychiatry, Rady Children’s Hospital, San Diego, California; 25 H. Ben Taub Department of Physical Medicine and Rehabilitation, Baylor College of Medicine, Houston, Texas; 26 Department of Psychology, Norwegian University of Science and Technology, Trondheim, Norway; 27 Clinic of Rehabilitation, St Olavs Hospital, Trondheim University Hospital, Trondheim, Norway; 28 NorHEAD-Norwegian Centre for Headache Research, Trondheim, Norway; 29 Department of Clinical Sciences, Murdoch Children’s Research Institute, Melbourne, Victoria, Australia; 30 Department of Paediatrics, The University of Melbourne, Melbourne, Victoria, Australia; 31 Department of Psychological Sciences, Texas Tech University, Lubbock; 32 South African Medical Research Council Unit on Risk and Resilience in Mental Disorders, Department of Psychiatry, Cape Town University, Cape Town, South Africa; 33 South African Medical Research Council Unit on Risk and Resilience in Mental Disorders, Neuroscience Institute, Cape Town University, Cape Town, South Africa; 34 Kennedy Krieger Institute, Baltimore, Maryland; 35 Department of Physical Medicine and Rehabilitation, Johns Hopkins University School of Medicine, Baltimore, Maryland; 36 Department of Pediatrics, Johns Hopkins University School of Medicine, Baltimore, Maryland; 37 Department of Psychology, Georgia State University, Atlanta; 38 Neuroscience and Mental Health Program, Hospital for Sick Children, Toronto, Ontario, Canada; 39 Physiology Department, University of Toronto, Toronto, Ontario, Canada; 40 Department of Pediatrics, University of Florida, Gainesville; 41 Department of Pediatrics, University of Utah School of Medicine, Salt Lake City; 42 Department of Neurology, University of Florida, Gainesville; 43 Imaging Genetics Center, Stevens Neuroimaging & Informatics Institute, Keck School of Medicine of the University of Southern California, Marina del Rey; 44 Department of Neurology, University of Southern California, Los Angeles; 45 Department of Pediatrics, University of Southern California, Los Angeles; 46 Department of Psychiatry, University of Southern California, Los Angeles; 47 Department of Radiology, University of Southern California, Los Angeles; 48 Department of Engineering, University of Southern California, Los Angeles; 49 Department of Ophthalmology, University of Southern California, Los Angeles; 50 Monash Biomedical Imaging, Monash University, Melbourne, Victoria, Australia

## Abstract

**Importance:**

Traumatic brain injury (TBI) is known to cause widespread neural disruption in the cerebrum. However, less is known about the association of TBI with cerebellar structure and how such changes may alter executive functioning.

**Objective:**

To investigate alterations in subregional cerebellum volume and cerebral white matter microstructure after pediatric TBI and examine subsequent changes in executive function.

**Design, Setting, and Participants:**

This retrospective cohort study combined 12 data sets (collected between 2006 and 2020) from 9 sites in the Enhancing Neuroimaging Genetics Through Meta-Analysis Consortium Pediatric TBI working group in a mega-analysis of cerebellar structure. Participants with TBI or healthy controls (some with orthopedic injury) were recruited from trauma centers, clinics, and institutional trauma registries, some of which were followed longitudinally over a period of 0.7 to 1.9 years. Healthy controls were recruited from the surrounding community. Data analysis occurred from October to December 2022.

**Exposure:**

Accidental mild complicated-severe TBI (msTBI) for those in the TBI group. Some controls received a diagnosis of orthopedic injury.

**Main Outcomes and Measures:**

Volume of 18 cerebellar lobules and vermal regions were estimated from 3-dimensional T1-weighted magnetic resonance imaging (MRI) scans. White matter organization in 28 regions of interest was assessed with diffusion tensor MRI. Executive function was measured by parent-reported scores from the Behavior Rating Inventory of Executive Functioning.

**Results:**

A total of 598 children and adolescents (mean [SD] age, 14.05 [3.06] years; range, 5.45-19.70 years; 386 male participants [64.5%]; 212 female participants [35.5%]) were included in the study, with 314 participants in the msTBI group, and 284 participants in the non-TBI group (133 healthy individuals and 151 orthopedically injured individuals). Significantly smaller total cerebellum volume (*d* = −0.37; 95% CI, −0.52 to −0.22; *P* < .001) and subregional cerebellum volumes (eg, corpus medullare; *d* = −0.43; 95% CI, −0.58 to −0.28; *P* < .001) were observed in the msTBI group. These alterations were primarily seen in participants in the chronic phase (ie, >6 months postinjury) of injury (total cerebellar volume, *d* = −0.55; 95% CI, −0.75 to −0.35; *P* < .001). Smaller cerebellum volumes were associated with higher scores on the Behavior Rating Inventory of Executive Functioning Global Executive Composite score (β = −208.9 mm^3^; 95% CI, −319.0 to −98.0 mm^3^; *P* = .008) and Metacognition Index score (β = −202.5 mm^3^; 95% CI, −319.0 to −85.0 mm^3^; *P* = .02). In a subset of 185 participants with longitudinal data, younger msTBI participants exhibited cerebellum volume reductions (β = 0.0052 mm^3^; 95% CI, 0.0013 to 0.0090 mm^3^; *P* = .01), and older participants slower growth rates. Poorer white matter organization in the first months postinjury was associated with decreases in cerebellum volume over time (β=0.52 mm^3^; 95% CI, 0.19 to 0.84 mm^3^; *P* = .005).

**Conclusions and Relevance:**

In this cohort study of pediatric msTBI, our results demonstrated robust cerebellar volume alterations associated with pediatric TBI, localized to the posterior lobe. Furthermore, longitudinal cerebellum changes were associated with baseline diffusion tensor MRI metrics, suggesting secondary cerebellar atrophy. These results provide further understanding of secondary injury mechanisms and may point to new opportunities for intervention.

## Key Points

**Question:**

Are there substantial alterations in cerebellar structure after traumatic brain injury (TBI) in children, and are they associated with changes in executive functioning?

**Findings:**

In this longitudinal cohort study of 598 children and adolescents, TBI was associated with widespread decreases in cerebellum volume, particularly in the posterior lobe, which were also associated with poorer executive function. Deficits in white matter organization, measured with diffusion tensor magnetic resonance imaging, were found to be associated with cerebellar disruption beyond general atrophy and injury severity.

**Meaning:**

These findings suggest that brain structural disruptions from TBI can evolve over time in regions associated with executive function that were not directly injured.

## Teaser

This cohort study analyzes alterations in subregional cerebellum volume and cerebral white matter microstructure and its association with executive function after pediatric traumatic brain injury (TBI).

## Introduction

Traumatic brain injury (TBI) is a leading cause of death and disability in children in the US^1^ and is associated with distinct characteristics due to age-related, developmental, anatomical, and physiological differences.^2^ Most pediatric TBI studies have ignored the cerebellum, exclusively targeting supratentorial brain areas that are assumed to be later-developing and/or more vulnerable to direct injury.^3^ Novel image processing tools allow for more fine-grained atlases and parcellation of the cerebellum, enabling charting of regional volume change over the life span.^4^ Developmental trajectories for cerebellar subregions are complex, with maturation peaking in the vermis and flocculonodular lobe at 5 years, the anterior lobe between 12 and 16 years (lobules I-V), and the posterior lobe in late adolescence and early adulthood (lobules VI-IX).^5^ Thus, many subregions of the cerebellum are in critical periods of development during adolescence, potentially making them especially vulnerable to TBI.

Motor functions of the cerebellum, including balance, coordination, motor learning, and body awareness, are well-established,^6^ but frontocerebellar brain systems also support executive functions,^7^ including multitasking,^8^ inhibition,^9^ working memory,^10^ social cognition,^11^ and emotional processing.^12^ Investigations of cerebellar injury in adult TBI,^13^ brain tumor,^14^ or stroke^15^ support these associations, but no prior studies have examined potential associations after pediatric TBI. Understanding cerebellum disruption may help address morbidity in pediatric TBI.

Although the cerebellum is less vulnerable than other areas of the brain to direct injury,^16^ decreases in white matter (WM) volume,^17^ reductions in fractional anisotropy (FA),^18^ functional dissociation,^19^ and hypoperfusion have been observed after TBI.^20^ One potential mechanism is connectomal diaschisis,^21^ whereby direct injury to the cerebrum propagates to the cerebellum via cerebellar structural and functional networks.^22,23^ Animal research supports this concept, with studies^24,25,26^ showing indirect alterations associated with disruption of corticocerebellar fibers. If this is also the case in humans, structural alterations would not be expected immediately postinjury but could develop over months.

We investigated volumetric cross-sectional differences and longitudinal changes in the cerebellum following pediatric complicated mild complicated-severe TBI (msTBI), further examining associations with WM microstructural organization and executive functioning. Enhancing Neuroimaging Genetics Through Meta-Analysis (ENIGMA) is a global research consortium achieving greater statistical power through coordinated processing of legacy data. Combining 12 cohorts from the ENIGMA Pediatric msTBI working group,^27,28,29,30,31,32,33,34,35,36,37,38,39^ we measured regional cerebellar volume in children and adolescents. A priori hypotheses were as follows: (1) cerebellar volume would be lower in individuals with msTBI vs non-TBI, (2) these disruptions would be most prominent furthest from the time of injury, and (3) smaller cerebellar volume would be associated with poorer executive functioning.

## Methods

### Study Design

The ENIGMA Pediatric msTBI working group^28,40,41^ brings together data from different sources to identify reliable neuroimaging biomarkers of injury and recovery. Initial hypotheses focused on cerebellar volumes, but these results motivated us to include available diffusion tensor imaging (DTI) data, hypothesizing that alterations in DTI would predate and be associated with changes in cerebellar volumes.

### Standard Protocol Approvals and Consent

Original studies were approved by the individual institutional review boards for each respective institution. All participants provided written or verbal informed assent, and parents provided written informed consent. All procedures in the current report followed the Strengthening the Reporting of Observational Studies in Epidemiology (STROBE) reporting guidelines for cohort studies.^42^

### Study Samples

We included 12 existing cohorts from 9 sites and included participants with TBI ranging between complicated mild (referred to as mild) to complicated severe TBI (Glasgow Coma Scale [GCS] score >12) with injury-related imaging abnormalities and participants without TBI. The non-TBI group included healthy children and children with orthopedic injury (recruitment and imaging details are shown in eTables 1 and 2 in Supplement 1). In line with prior publications,^27^ we divided msTBI participants into 3 postinjury windows: (1) acute and subacute (magnetic resonance imaging [MRI] within 7 weeks postinjury), when pathology such as intracerebral hemorrhage and edema are prominent; (2) postacute (MRI 8 weeks to 6 months postinjury), where secondary injuries such as regional atrophy and microstructural alterations become apparent; and (3) chronic (MRI more than 6 months postinjury), when some recovery and/or atrophy continues, but the brain is more neurologically stable.^27^ Exact boundaries were based on published data and natural break points within data sets.

### Image Acquisition, Processing, and Quality Control

Methods are reviewed here with additional detail in eMethods in Supplement 1. Raw 3-dimensional T1-weighted MR images were processed using the ENIGMA Cerebellum Pipeline, based on Automatic Cerebellum Anatomical Parcellation using U-Net with Locally Constrained Optimization (ACAPULCO version 0.2.1; Johns Hopkins University).^4,43,44^ Image processing, segmentation, and quality review occurred at the University of Utah. The cerebellum was segmented into 28 subregions (eFigure 1 in Supplement 1). Segmentations were visually quality-checked and statistical outliers (>3 SDs) for each region of interest (ROI) were excluded (eTable 3 in Supplement 1). Scans were checked for cerebellar lesions (visible on T1-weighted scans [44 scans]). We examined volume of the total cerebellum, corpus medullare, 5 vermal regions, and 11 lateralized lobules (left-right averaged), for a total of 18 cerebellar ROIs. We also conducted analyses for the subset of participants with longitudinal data (2 time points) through ACAPULCO version 0.3.0, further optimized for longitudinal analysis.^4,45^

DTI data for 28 ROIs were processed as detailed in our previous publication.^27^ Briefly, we used the ENIGMA-DTI pipeline^46^, a modified tract-based spatial statistics approach^47^ resulting in FA and other metrics averaged within ROIs from the Johns Hopkins University atlas. Of the 12 cohorts, 10 collected DTI data (parameters in eTable 4 in Supplement 1).

### Neurobehavioral Measures

As a retrospective analysis of multiple cohorts, there was variability in the neurobehavioral scales administered. We limited our analyses to inventories most common across cohorts. Work is ongoing in the ENIGMA Brain Injury working group to harmonize scales within the same domain.^48^

The Behavior Rating Inventory of Executive Function (BRIEF) is a widely used parent and informant questionnaire measuring executive functioning in children.^49^ Parents respond to questions about their child’s behaviors, resulting in 3 age-adjusted scores. The Behavioral Regulation Index (BRI) measures cognitive abilities, such as inhibition, task shifting, emotional control, and self-monitoring, and the Metacognition Index (MCI) measures initiation, working memory, planning and organizing, and task monitoring. The Global Executive Composite (GEC) is an overarching summary score of executive functioning. For each of these scores, higher scores indicate greater executive dysfunction. Details on scores in our sample are in eMethods in Supplement 1.

### Statistical Analysis

Linear mixed-effects models were conducted in R statistical software version 3.1.3 (R Project for Statistical Computing) with nlme.^50^ Random effects (intercept) controlled for site and participant. All analyses covaried for age, sex, and intracranial volume (ICV). We computed Cohen *d* effect sizes with 95% CIs and unstandardized β values for continuous variables, using a modified Bonferroni correction for multiple comparisons (eMethods and eAppendix in Supplement 1).^51^ This method accounts for the associations between regions tested, calculating an effective number of variables (V_eff_) and scaling appropriately: *P* = .05/V_eff_. All *P* values reported were adjusted for multiple comparisons unless otherwise specified, with a 2-sided *P* < .0045 indicating statistical significance.^51^ A flowchart of analyses is in eFigure 2 in Supplement 1. Data analysis occurred from October to December 2022.

#### Group Comparisons

Primary analyses compared msTBI with non-TBI including all postinjury windows. One cohort lacked a non-TBI group and was omitted from group analyses. Further, we examined differences in total cerebellum volume change in a subset of participants (75 participants), covarying for scan interval and time since injury (TSI) at first scan.

Secondary sensitivity analyses were conducted covarying for TSI and excluding acute patients given that acute pathology could influence neuroimaging metrics. We also separated cohorts on the basis of non-TBI population (healthy vs orthopedic injury), by severity, and by injury phase (acute, postacute, and chronic). We repeated analyses excluding scans with cerebellar lesions visible on T1-weighted images (73 excluded participants).

#### Supplemental Analyses

We examined potential interactions with group, including age and sex. Within msTBI, we examined potential interactions between age at injury and TSI, age at injury and GCS, and GCS and TSI. Within msTBI, we investigated associations with age at injury, TSI, and GCS, covarying for age, sex, and ICV. Within msTBI, we investigated associations of cerebellum volumes with BRIEF scores.

#### Exploratory Multimodal Analyses

Based on the primary group comparison results, we conducted exploratory multimodal analyses including DTI metrics. We examined associations of FA with cerebellar volumes in the msTBI group collected concurrently (252 volumes) covarying for age, sex, ICV, and GCS. Furthermore, we explored the estimation value of FA, examining associations of FA with total cerebellum volume change. Because the multimodal analyses were exploratory, we used an uncorrected threshold of *P* < .05 and reported uncorrected *P* values.

## Results

The 12 cohorts resulted in a study pool of 598 participants (mean [SD] age, 14.05 [3.06] years; range, 5.45-19.70 years; 386 male participants [64.5%]; 212 female participants [35.5%]) including 314 participants in the msTBI group, and 284 participants in the non-TBI group (133 healthy individuals and 151 orthopedically injured individuals) (Table 1). Within the msTBI group (mean [SD] age at injury, 13.0 [3.6] years), we had 67 acute scans, 122 postacute scans, and 224 chronic scans. Of the 12 cohorts, 7 were from longitudinal studies, and 5 were from cross-sectional studies, generating 783 scans (185 with longitudinal data). The mean (SD) interval between scans was 1.1 (0.3) years (range, 0.7-1.9 years). Main results are summarized below, with additional results in eResults in Supplement 1.

**Table 1.** Cohort Demographics

Characteristic, No.	Cohort																							Total
	RAPBI	Pilot-RAPBI		NCH		KKI		LLU		DU 1		DU 2		BCM 1^a^		BCM 2^a^		BCM 3^a^		MCRI		UT Houston^b^		
Participants																								
Total	109		22		53		42		52		44		49		99		32		31		22		43	598
msTBI group	53		13		29		29		21		18		22		50		15		22		22		20	314
Comparison group	56		9		24		13		31		26		27		49		17		9		0		23	284
Sex																								
Male	72		14		37		27		34		20		23		71		21		21		17		29	386
Female	37		8		16		15		18		24		26		28		11		10		5		14	212
Age, y																								
Mean (SD)	15.61 (2.79)		16.14 (1.86)		11.80 (2.38)		14.57 (2.43)		12.89 (3.45)		14.34 (2.80)		14.60 (2.73)		13.53 (2.83)		14.56 (2.62)		15.48 (2.34)		11.13 (2.86)		12.70 (2.41)	14.05 (3.06)
Range	8.40 to 19.70		12.10 to 18.57		8.16 to 16.52		8.12 to 18.98		5.45 to 17.78		8.52 to 19.00		8.52 to 18.97		7.44 to 18.70		10.67 to 19.40		10.61 to 18.51		5.83 to 16.83		8.16 to 16.91	5.45 to 19.70
TSI range, wk^c^																								
Period 1	3.9-36.3		11.7-36.2		58.7-42.4		4.1-14.6		1.0-2.6		15.6-56.2		NA		11.7-28.8		0.14-15.4		2.4-60.0		4.5-37.6		4.1-18.3	NA
Period 2	49.2-82.7		59.1-68.6		NA		51.9-76.0		48.1-62.1		NA		NA		49.6-117.8		NA		NA		99.8-124.0		55.8-63.0	NA
Scans																								
Total	158		24		53		55		100		44		49		134		32		31		43		60	783
Longitudinal	49		2		NA		13		48		NA		NA		35		NA		NA		21		17	185

Abbreviations: BCM, Baylor College of Medicine; DU; Deakin University; KKI, Kennedy-Krieger Institute; LLU, Loma Linda University; MCRI, Murdoch Children’s Research Institute; msTBI, mild complicated-severe traumatic brain injury; NA, not applicable; NCH, Nationwide Children’s Hospital; RAPBI, Recovery After Pediatric Injury; TSI, time since injury; UT, University of Texas.

^a^ Indicates sites with comparison groups consisting of individuals with orthopedic injury.

^b^ UT Houston had both orthopedic individuals and healthy controls in the comparison group.

^c^ Studies with 2 time periods are longitudinal studies.

### Group Comparisons

#### Cross-Sectional Comparisons

**Figure 1.**
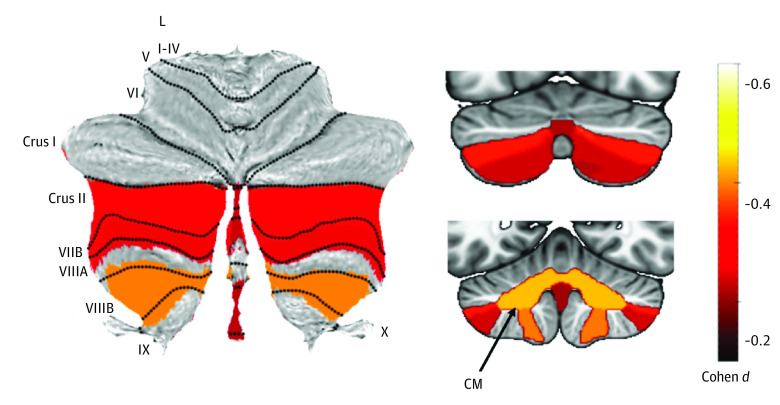
Primary Group Comparison Atlas-based effect size (Cohen *d*) maps and Montreal Neurological Institute-based coronal slices (top, y-axis = −72; bottom, y = −54) of the significant between-group differences for children with mild complicated severe traumatic brain injury vs controls. CM indicates corpus medullare; L, lobule.

Including data from all time points, the msTBI group had significantly smaller volumes for total cerebellum (*d* = −0.37; 95% CI, −0.52 to −0.22; *P* < .001), corpus medullare (*d* = −0.43; 95% CI, −0.58 to −0.28; *P* < .001), Crus II, lobules VIIB and VIIIB, and vermis VII and IX, compared with the non-TBI group. Results are summarized in Figure 1 and Table 2. Removing participants with visible cerebellar lesions yielded similar results, although the vermal effect sizes were no longer significant.

**Table 2.** Primary Group Comparison

Region and subregion	Cohen *d* value (95% CI)	*P* value^a^	Adjusted *P* value
Total volume	−0.37 (−0.52 to −0.22)	<.001	<.001
Corpus medullare	−0.43 (−0.58 to −0.28)	<.001	<.001
Anterior lobe			
Lobule I-III	−0.08 (−0.23 to 0.07)	.32	.98
Lobule IV	0.02 (−0.13 to 0.17)	.75	>.99
Lobule V	−0.18 (−0.33 to −0.03)	.02	.17
Posterior lobe			
Lobule VI	−0.18 (−0.34 to −0.02)	.03	.26
Crus I	−0.23 (−0.41 to −0.05)	.01	.12
Crus II	−0.32 (−0.49 to −0.16)	<.001	.001
Lobule VIIB	−0.25 (−0.41 to −0.10)	.002	.02
Lobule VIIIA	−0.06 (−0.22 to 0.11)	.50	>.99
Lobule VIIIB	−0.39 (−0.56 to-0.22)	<.001	<.001
Lobule IX	−0.17 (−0.33 to −0.01)	.03	.31
Flocculonodular lobe, lobule X	0.00 (−0.15 to 0.15)	.98	>.99
Vermis			
VI	0.06 (−0.09 to 0.21)	.41	>.99
VII	−0.23 (−0.38 to −0.08)	.003	.03
VIII	−0.12 (−0.27 to 0.03)	.12	.76
IX	−0.22 (−0.37 to −0.07)	.004	.047
X	−0.19 (−0.34 to −0.04)	.01	.13

^a^ The threshold for significance for the raw *P* values is *P* < .0045.

**Figure 2.**
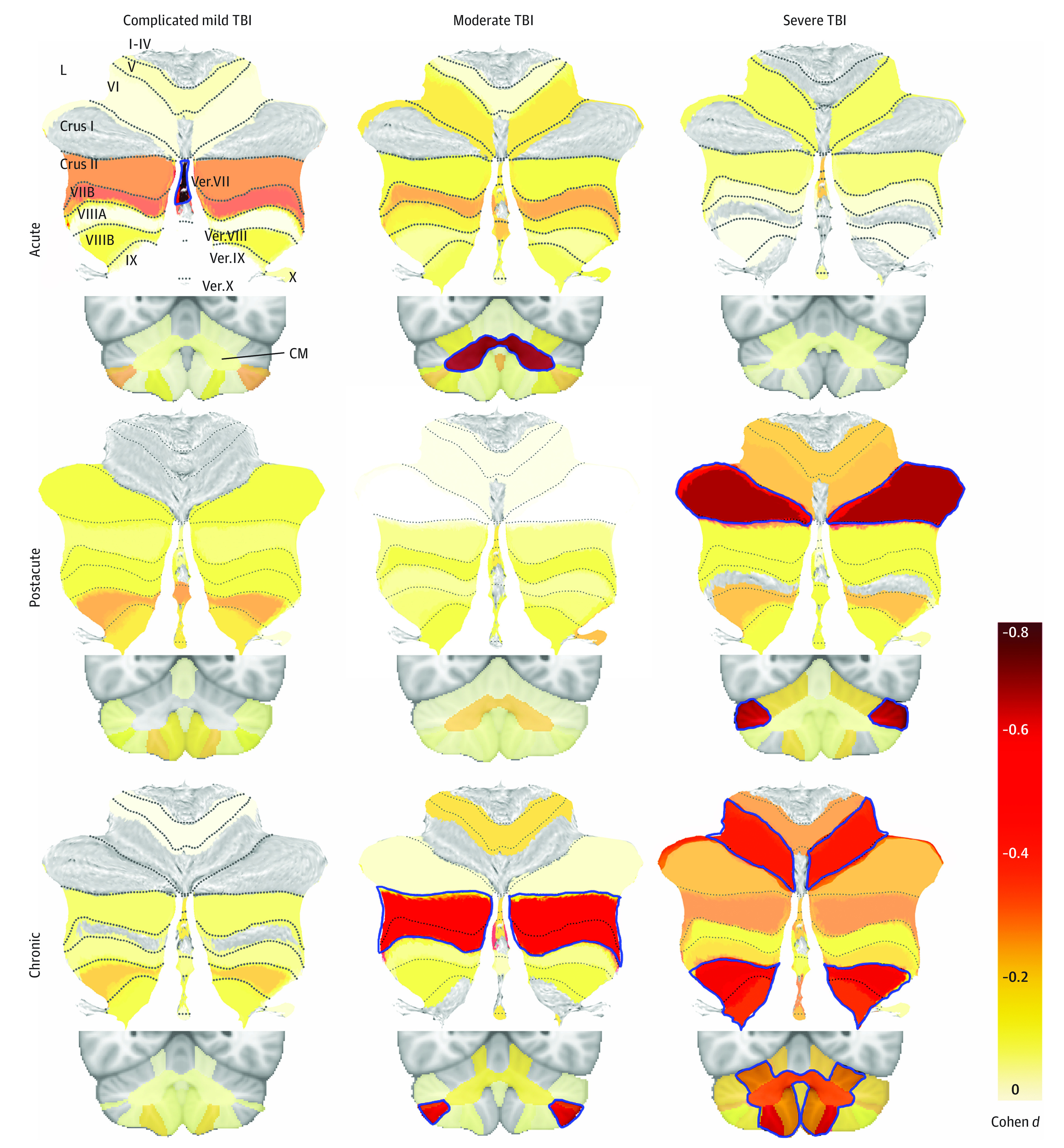
Severity and Chronicity Analyses Atlas-based effect size (Cohen *d*) maps and Montreal Neurological Institute–based coronal slices are shown for group comparisons separated by severity (columns) and chronicity (rows). Lobules and vermal regions (Vermis [Ver.] VII-X) are labeled in the top left on the spatially unbiased infratentorial template flatmap. The corpus medullare (CM) is shown in the coronal slices. The color corresponds to the effect size, according to the color bar, with dark red for the largest effect sizes. Nonsignificant effect sizes are shown at 50% opacity, whereas significant ones are not opaque and outlined in blue. The number of traumatic brain injury (TBI) and non-TBI participants for each comparison are as follows: 12 participants with acute complicated mild TBI, 7 participants with acute moderate TBI, and 25 patients with acute severe TBI, who were each compared with 82 non-TBI participants; 26 patients with postacute complicated mild TBI, 12 patients with postacute moderate TBI, and 43 patients with postacute severe TBI, who each compared with 143 non-TBI participants; and 32 patients with chronic complicated mild TBI, 18 patients with chronic moderate TBI, and 71 patients with chronic severe, who were each compared with 209 non-TBI participants. No TBI participant was included twice in any of the 9 subanalyses, but non-TBI participants were included across multiple comparisons. Only negative effect sizes are shown; positive effect sizes were not significant and are not included.

Separating participants on the basis of TSI, group differences were predominantly associated with participants in the chronic phase (total cerebellar volume, *d* = −0.55; 95% CI, −0.75 to −0.35; *P* < .001) with no postacute difference surviving multiple comparisons correction and a significantly smaller vermis VII in the acute phase (eTable 5 in Supplement 1). Separated by severity, we found group differences primarily in moderate and severe TBI (eTable 6 in Supplement 1). There were no significant differences between complicated mild TBI and non-TBI. The above severity and chronicity analyses were run separately for 6 different comparisons (3 for each group). To visualize effect sizes, we also ran these analyses combined, for 9 different comparisons. These results are tiled by severity and chronicity in Figure 2. Residuals of the model fit were assessed to test model assumptions. No evidence of residual bias or correlation with covariates was found. However, the residuals were found to be nonnormally distributed, owing to a small number of symmetric residual outliers (35 of 697 residuals [5%]) above or below the 1.5 IQR Tukey rule limits. Excluding these outliers above and below 1.5 IQR, the remaining 95% of residuals were found to be normally distributed.

#### Longitudinal Comparisons

Total cerebellum volume growth was significantly smaller in the msTBI group vs the non-TBI group (75 participants; *d* = −0.55; 95% CI, −1.02 to -0.09; *P* = .02) (eFigure 3 in Supplement 1). This outcome persisted when excluding msTBI participants with cerebellar lesions (73 participants; *d* = −0.55; 95% CI, −1.02 to −0.07; *P* = .02), and when covarying for changes in total brain volume (*d* = −0.62; 95% CI, −1.10 to −0.15; *P* = .01). In the msTBI group, total cerebellum volume decreased in 20 participants and increased in 25 participants. Within the msTBI group, changes in total cerebellum volume were associated with age at injury, with more volume decreases in patients who were injured at a younger age (β = 0.0052 mm^3^; 95% CI, 0.0013 to 0.0090 mm^3^; *P* = .01) whereas older participants experienced slower growth rates (eFigure 4 in Supplement 1).

Post hoc tests examined potential confounders such as acute-phase pathology, comparison group type, and attention-deficit/hyperactivity disorder (ADHD). Results were consistent with our primary models. These are summarized in eResults and eTables 7 and 8 in Supplement 1.

### Supplemental Analyses

There was a significant interaction between TSI and GCS for total cerebellum, whereby participants with higher GCS scores showed increased volume with further TSI (eResults, eTable 9, and eFigure 5 in Supplement 1). Within msTBI, there were no significant associations of TSI with GCS, or age at injury (controlling for age at scan). Total cerebellum volume was negatively associated with the BRIEF MCI (β = −202.5 mm^3^; 95% CI, −319.0 to −85.0 mm^3^; *P* = .02) and GEC (β = −208.9 mm^3^; 95% CI, −319.0 to −98.0 mm^3^; *P* = .008) scores in msTBI, such that smaller cerebellar volumes were associated with greater executive dysfunction (Table 3).

**Table 3.** BRIEF Score Associations

Region and subregion	BRIEF Behavioral Regulation Index			BRIEF Metacognition Index			BRIEF Global Executive Composite		
	*P* value^a^	Adjusted *P* value	β-Value (95% CI)	*P* value^a^	Adjusted *P* value	β-Value (95% CI)	*P* value^a^	Adjusted *P* value	β-Value (95% CI)
Total volume	.005	.06	−152.9 (−253.7 to −52.0)	.002	.02	−202.5 (−319.5 to −85.5)	<.001	.008	−208.9 (−319.4to −98.5)
Corpus medullare	.31	.98	−11 (−32.0 to 10.1)	.08	.60	−23.4 (−49.1 to 2.4)	.08	.60	−22.1 (−46.5 to 2.3)
Anterior lobe									
Lobule I-III	.23	.94	−1.5 (−3.8 to 0.9)	.02	.20	−3.0 (−5.4 to −0.6)	.08	.60	−2.2 (−4.6 to 0.2)
Lobule IV	.05	.41	−5.6 (−11.0 to −0.2)	.005	.05	−8.7 (−14.4 to −3.0)	.007	.07	−8.2 (−13.8 to −2.6)
Lobule V	.03	.27	−6.1 (−11.4 to −0.8)	.02	.16	−7.1 (−12.7 to −1.6)	.02	.16	−7.1 (−12.5 to −1.6)
Posterior lobe									
Lobule VI	.34	.99	−5.9 (−18.2 to 6.3)	.06	.48	−13.7 (−27.2 to −0.2)	.16	.85	−9.6 (−23.0 to 3.7)
Crus I	.02	.22	−27.9 (−50.2 to −5.6)	.09	.66	−21.1 (−45.0 to 2.7)	.03	.26	−28.0 (−51.2 to −4.8)
Crus II	.54	>.99	−4.5 (−19.0 to 10.0)	.08	.58	−14.7 (−30.4 to 1.1)	.19	.90	−10.7 (−26.6 to 5.2)
Lobule VII B	.76	>.99	2.0 (−10.5 to 14.4)	.12	.76	−10.7 (−24.0 to 2.5)	.19	.90	−9.0 (−22.5 to 4.4)
Lobule VIIIA	.31	.98	−6.4 (−18.7 to 6.0)	.81	>.99	1.6 (-11.4 to 14.6)	.95	>.99	−0.4 (−13.4 to 12.6)
Lobule VIIIB	.62	>.99	−1.6 (−7.9 to 4.7)	.58	>.99	−2.1 (−9.6 to 5.4)	.41	>.99	−3.0 (−10.3 to 4.2)
Lobule IX	.13	.78	−4.3 (−9.9 to 1.2)	.16	.85	−5.0 (−11.8 to 1.8)	.32	.99	−3.4 (−10.0 to 3.3)
Flocculonodular lobe, lobule X	.19	.90	0.7 (−0.3 to 1.7)	.32	>.99	0.6 (−0.5 to 1.7)	.53	>.99	0.3 (−0.7 to 1.4)
Vermis									
VI	.12	.76	1.7 (−1.3 to 4.7)	.96	>.99	0.5 (−2.6 to 3.7)	.85	>.99	1.1 (−2.0 to 4.2)
VII	.86	>.99	−1.5 (−3.3 to 0.3)	.81	>.99	0.0 (−2.1 to 2.0)	.50	>.99	−0.2 (−2.2 to 1.8)
VIII	.78	>.99	0.3 (−3.0 to 3.6)	.86	>.99	0.4 (−3.2to 4.1)	.73	>.99	1.2 (−2.3 to 4.7)
IX	.37	.99	−0.2 (−1.8 to 1.4)	.95	>.99	0.2 (−1.6 to 1.9)	.84	>.99	0.3 (−1.4 to 2.0)
X	.27	.97	0.3 (−0.3 to 0.9)	.75	>.99	0.0 (−0.7 to 0.7)	.47	>.99	0.1 (−0.6 to 0.7)

Abbreviations: BRIEF, Behavior Rating Inventory of Executive Functioning.

^a^ The threshold for significance for the raw *P* values is *P* < .0045.

### Exploratory Multimodal MRI Analyses

In the exploratory multimodal analysis, there were 32 participants from the msTBI group with high-quality DTI data at time point 1, high-quality cerebellar segmentations at both time points, and data for all necessary covariates. Among participants with msTBI, there were significant cross-sectional associations of total cerebellum volume with FA in central WM ROIs (eTable 10 and eFigure 6 in Supplement 1). This finding was significant when covarying for GCS, suggesting that the association of cerebellar volume with FA in the cerebrum is not dependent on TBI severity. FA at baseline was also significantly associated with longitudinal changes in total cerebellum volume (β=0.52 mm^3^; 95% CI, 0.19 to 0.84 mm^3^; *P* = .005) (further regional details in eResults in Supplement 1). We covaried for interval, TSI, GCS, and percentage change in ICV, indicating that the association of baseline FA with secondary cerebellar changes again were associated with injury severity or overall atrophy. The only significant cross-sectional or longitudinal associations of FA with total cerebellum volume in the non-TBI group were the cerebellar peduncles.

## Discussion

In what is, to our knowledge, the largest MRI cohort study of pediatric msTBI, we found smaller total cerebellum volume which was associated with changes in the posterior lobe. Volume reductions were most prominent in patients with more severe injuries and those at least 6 months postinjury and were independent from general injury severity and global atrophy, suggesting that volumetric changes in the cerebellum may be due to a secondary injury process. This secondary injury hypothesis was substantiated with longitudinal analyses incorporating multimodal MRI. Our results indicate that regions not directly impacted by injury cannot be assumed to be spared, and that these late-developing disruptions are associated with executive function. Finally, longitudinal analyses showed cerebellar atrophy in the youngest participants, which may partially explain generally worse outcomes; however, we were unable to consider other factors confounded with age such as mechanism of injury, which may also be associated outcomes.

### Cerebellum Development

Developmental trajectories of the cerebellum are complex, with peak maturation ranging between age 5 years to early adulthood, generally with the vermis and anterior lobe maturing earliest; therefore, posterior lobular cerebellar regions were likely immature in our sample (mean [SD] age at injury, 13.0 [3.6] years), possibly increasing their vulnerability. Plasticity during development may lead to faster recovery but also increased susceptibility to disruption.^52^ However, the posterior cerebellum may simply be particularly susceptible to disruption, perhaps due to connectivity with the prefrontal cortex.^53,54,55^ Two recent examinations in ENIGMA working groups have shown volume deficits among adults with posttraumatic stress disorder and epilepsy, primarily in the posterior lobe.^44,56^ Future analyses with expanded age ranges and multimodal MRI data may further disentangle potential sources of susceptibility.

### Potential Sources of Cerebellar Vulnerability

The frontocerebellar networks supporting the cerebellum’s role in cognitive function may be a source of indirect injury. The posterior lobe, where the greatest volumetric deficits in msTBI were found, is of particular interest given that it is larger in primates compared with other mammals^57^ and has been shown during phylogenetic expansion to mirror the frontal cortex.^54,55^ In the absence of direct injury, atrophy in the cerebellum may be associated with secondary injury processes such as connectomal diaschisis. Effect sizes were largest in the corpus medullare, where the deep cerebellar nuclei (the terminus for many cortical projections) are located. Higher resolution data may determine whether the structural connectivity of the cerebellum is associated with increased vulnerability. We found significant differences in the chronic phase of injury, with few differences in the acute or postacute phases, suggesting that atrophy occurs months after the initial injury. We substantiated these findings with a secondary longitudinal analysis and found greater total cerebellum volume decreases in msTBI. Additionally, lower FA of multiple central WM regions was associated with slower growth in the cerebellum, even when controlling for injury severity and change in total brain volume, indicating that they are not simply associated with general neuropathology postinjury. In the non-TBI group, only FA of the cerebellar peduncles was associated with cerebellum volume, suggesting that these are unique to the context of injury. The contribution of other acute pathology, such as lesions, is not clear, and further work on the interconnected structural and functional networks of the cerebellum is necessary.

### Potential Confounders

One important confounding variable is the occurrence of preexisting psychiatric disorders within our sample. In particular, ADHD is associated with increased risk of TBI^58^ and is also associated with smaller cerebellum volume.^59^ We, thus, conducted a secondary analysis only using data from sites that excluded participants with ADHD and found consistent results.

### Limitations

This study has limitations. Despite our large sample size, differences among recruitment criteria, scan parameters, and behavioral measures between sites limit the power of some analyses. Variability across sites at the time of testing and scanning postinjury may have limited our results because the first year after injury is particularly dynamic.^27^ We established postinjury intervals and conducted analyses within each interval to better understand this phenomenon. However, physiologic changes occur along a continuum, and not in discrete periods. There were differences in sample size within the postinjury intervals, and only a subset had longitudinal data. Although the multisite design led to variability, it also resulted in what is, to our knowledge, the largest pediatric msTBI MRI sample to date, demonstrating the utility of ENIGMA to support analyses that might otherwise be underpowered. Additionally, tract-based spatial statistics is an ROI-based approach limiting our ability to fully attribute our results to specific tracts. Further mapping of the structural connectome using tractography may provide more detail.

## Conclusion

In this cohort study, cerebellum volume was significantly smaller in patients with msTBI, and was most pronounced in the later-developing posterior lobe. Furthermore, these volumetric alterations were associated with poorer executive functioning. Longitudinal and multimodal results indicated that indirect cerebellar injury may be associated with early injury-related disruptions in cerebral WM microstructure, beyond general injury and atrophy. These results suggest ongoing neural processes postinjury that result in cerebellar changes, which in turn contribute to executive function deficits, and highlight the importance of continuing to monitor patients long term as new clinical complaints emerge. Future research incorporating lesion mapping and structural connectivity will help understand the mechanisms of spreading disruption and potentially identify additional opportunities for intervention that may leverage developmental neuroplasticity.

## Notes

Supplement 1. **eTable 1.** Cohort Details **eTable 2.** Scan and Protocol Details
eMethods
**eFigure 1.** ACAPULCO Segmentation **eTable 3**. Quality Control Exclusions by ROI and Site **eTable 4.** DTI Protocol Details **eAppendix.** Li and Ji Modified Bonferroni Correction. **eFigure 2.** Flowchart of Analyses
eResults
**eTable 5.** Group Comparisons in Different Phases of Injury **eTable 6.** Group Comparisons With Different TBI Severity **eFigure 3.** Group Differences in Longitudinal Changes in Cerebellum Volume **eFigure 4.** Association Between Age at Injury and Percent Change in Total Cerebellum Volume for the Subset of TBI Participants With Longitudinal Data Available With Outliers Winsorized to 3SD **eTable 7.** Supplemental Group Comparisons **eTable 8.** Group Comparisons With Different Control Groups **eTable 9.** Interactions **eFigure 5.** Interaction Between Time Since Injury and Injury Severity **eTable 10.** Cross-Sectional and Longitudinal Associations Between FA and Total Cerebellum Volume in the TBI Group **eFigure 6.** White Matter Regions Associated With Longitudinal Changes in Total Cerebellum Volume in the TBI Group

Supplement 2.
Data Sharing Statement
