# Supplementary material for: Multimodal Analysis of Secondary Cerebellar Alterations After Pediatric Traumatic Brain Injury
Source: JAMA Netw Open. 2023 Nov 15;6(11):e2343410. doi: 10.1001/jamanetworkopen.2023.43410 (PMC10652147; doi:10.1001/jamanetworkopen.2023.43410)
Supplement: Supplement 1. — eTable 1. Cohort Details eTable 2. Scan and Protocol Details eMethods eFigure 1. ACAPULCO Segmentation eTable 3. Quality Control Exclusions by ROI and Site eTable 4. DTI Protocol Details eAppendix. Li and Ji Modified Bonferroni Correction. eFigure 2. Flowchart of Analyses eResults eTable 5. Group Comparisons in Different Phases of Injury eTable 6. Group Comparisons With Different TBI Severity eFigure 3. Group Differences in Longitudinal Changes in Cerebellum Volume eFigure 4. Association Between Age at Injury and Percent Change in Total Cerebellum Volume for the Subset of TBI Participants With Longitudinal Data Available With Outliers Winsorized to 3SD eTable 7. Supplemental Group Comparisons eTable 8. Group Comparisons With Different Control Groups eTable 9. Interactions eFigure 5. Interaction Between Time Since Injury and Injury Severity eTable 10. Cross-Sectional and Longitudinal Associations Between FA and Total Cerebellum Volume in the TBI Group eFigure 6. White Matter Regions Associated With Longitudinal Changes in Total Cerebellum Volume in the TBI Group [file jamanetwopen-e2343410-s001.pdf]

Supplemental Online Content

Keleher F, Lindsey HM, Kerestes R, et al. Multimodal analysis of secondary cerebellar alterations after pediatric traumatic brain injury. *JAMA Netw Open*. 2023;6(11):e2343410.
doi:10.1001/jamanetworkopen.2023.43410

eTable 1. Cohort Details
eTable 2. Scan and Protocol Details
eMethods
eFigure 1. ACAPULCO Segmentation
eTable 3. Quality Control Exclusions by ROI and Site
eTable 4. DTI Protocol Details
eAppendix. Li and Ji Modified Bonferroni Correction.
eFigure 2. Flowchart of Analyses
eTable 5. Group Comparisons in Different Phases of Injury
eTable 6. Group Comparisons With Different TBI Severity
eFigure 3. Group Differences in Longitudinal Changes in Cerebellum Volume
eFigure 4. Association Between Age at Injury and Percent Change in Total Cerebellum Volume for the Subset of TBI Participants With Longitudinal Data Available With Outliers Winsorized to 3SD
eResults
eTable 7. Supplemental Group Comparisons
eTable 8. Group Comparisons With Different Control Groups
eTable 9. Interactions
eFigure 5. Interaction Between Time Since Injury and Injury Severity
eTable 10. Cross-Sectional and Longitudinal Associations Between FA and Total Cerebellum Volume in the TBI Group
eFigure 6. White Matter Regions Associated With Longitudinal Changes in Total Cerebellum Volume in the TBI Group

This supplemental material has been provided by the authors to give readers additional information about their work.

This supplemental material has been provided by the authors to give readers additional information about their work.

© 2023 Keleher F et al. *JAMA Network Open*

eTable 1. Cohort Details

For each cohort, the recruitment location is shown, along with inclusion and exclusion criteria. * = cohorts with OI controls.

Cohort	Recruitment	Inclusion criteria	Exclusion criteria
RAPBI	Los Angeles County Pediatric Intensive Care Units (PICUs). Physicians identified potential participants who were contacted by a study representative. Controls recruited from community.	1) For TBI group: non-penetrating msTBI (moderate-severe) (intake or post-resuscitation Glasgow Coma Scale (GCS) score between 3 and 12); 2) 8-19 years of age; 3) right-handed; 4) normal visual acuity or vision corrected with contact lenses/eyeglasses; and 5) English skills sufficient to understand instructions and be familiar with common words (the neuropsychological tests used in this study presume competence in English).	1) history of neurological illness, such as prior msTBI, brain tumor or severe seizures; 2) motor deficits that prevent the subject from being examined in an MR scanner (e.g., spasms, movement disorder); 3) history of psychosis, ADHD, Tourette's Disorder, learning disability, mental retardation, autism or substance abuse. These conditions are associated with cognitive impairments that might overlap with those caused by TBI. MRI contraindication was also an exclusion criterion.
Pilot-RAPBI	Los Angeles County Pediatric Intensive Care Units (PICUs). Physicians identified potential participants who were contacted by a study representative. Controls recruited from the community.	1) For TBI group: non-penetrating msTBI (moderate-severe) (intake or post-resuscitation Glasgow Coma Scale (GCS) score between 3 and 12); 2) 8-19 years of age; 3) right-handed; 4) normal visual acuity or vision corrected with contact lenses/eyeglasses; and 5) English skills sufficient to understand instructions and be familiar with common words (the neuropsychological tests used in this study presume competence in English).	1) history of neurological illness, such as prior msTBI, brain tumor or severe seizures; 2) motor deficits that prevent the subject from being examined in an MR scanner (e.g., spasms, movement disorder); 3) history of psychosis, ADHD, Tourette's Disorder, learning disability, mental retardation, autism or substance abuse. These conditions are associated with cognitive impairments that might overlap with those caused by TBI. MRI contraindication was also an exclusion criterion.

© 2023 Keleher F et al. *JAMA Network Open*

NCH*	TBI and trauma controls were recruited by mail from institutional trauma registries in Ohio. Interested participants were screened by phone.	1) hospitalized for at least one night for moderate to severe TBI (post-resuscitation GCS score between 3 and 12) or fracture (control OI group); 2) 8-15 years of age; 3) injured 1-4 years prior.	1) a history of previous TBI requiring medical treatment (i.e., prior to the target injury); 2) premorbid neurological disorder or mental retardation, or full-time special education placement at school; 3) injury as a result of child abuse or assault; 4) a history of severe psychiatric disorder requiring hospitalization; 5) sensory or motor impairment that precludes completion of study measures; 6) primary language other than English; 7) any contraindication to MRI; 8) refusal by the child's school to participate in the school visit.
**Cohort**	**Recruitment**	**Inclusion criteria**	**Exclusion criteria**
Kennedy-Krieger	Potential participants with TBI were identified from current or historical inpatient or outpatient rehabilitation care at Kennedy Krieger Institute. Controls recruited through word of mouth and community postings.	1) aged 8-18 years of age at time of all study visits; 2) Child and parent are conversant in English, as determined by their ability to understand and participate in English conversations to review the screening form (parent) and consent form (parent and child) 3) For TBI group: moderate or severe traumatic brain injury as defined by first Glasgow Coma Score of 12 or lower upon admission to first emergency room OR post-traumatic amnesia lasting longer than one hour OR alteration in consciousness lasting longer than 15 minutes OR injury-related intracranial abnormality on brain CT or MRI.	1) children in foster care, 2) penetrating TBI or open TBI (evidenced by dural tear), 3) inability to complete laboratory tasks due to cognitive or motor deficits or uncorrected visual impairment (including red/green colorblindness), 4) ongoing post-traumatic amnesia at the time of first study evaluation or 5) pre-injury diagnosis of mental retardation, psychiatric disorder, or developmental disorder other than ADHD with or without Oppositional Defiant Disorder (ODD). MRI contraindication was also an exclusion criterion.
LLU	TBI patients were recruited from hospitals. Control participants were recruited from Loma Linda University Health System pediatric clinics.	1) age between 4 and 18 years at the time of injury; 2) absence of previous brain injury, neurological disorders, drug or alcohol abuse, or MRI contraindications, including dental braces; 3) moderate-to-severe TBI with Glasgow Coma Scale (GCS) scores between 3 and 12 or complicated mild (cMild) TBI (GCS 13–15) if hemorrhage was detected on an acute brain imaging (CT) exam. Control subjects who met criteria 1 and 2 were recruited from our pediatric clinics and were scanned without sedation.	1) MRI contraindications; 2) neurological disorders; 3) previous brain trauma or alcohol or drug abuse, 4) history of significant cognitive deficit (i.e. pervasive developmental disorders (PDD))

© 2023 Keleher F et al. *JAMA Network Open*

Deakin University (1)		All moderate to severe TBI patients were assessed at least six months post-injury, when neurological recovery was stabilized. Participants were excluded if they had pre-existing developmental or intellectual disabilities, a progressive disease, or were taking medication.	All control subjects were screened to ensure that they had no history of neurological damage.
Deakin University (2)	The participants were part of a larger-scale cognitive training study in pediatric TBI	Inclusion criteria for patients were as follows: (1) Age at injury: 10–17 years; (2) Injuries classified as moderate to severe using the Mayo Classification System; and (3) In the chronic stage of injury at the time of assessment (1–5 years post injury). All participants met magnetic resonance imaging (MRI) safety criteria (i.e., no metal implants or orthodontic braces) and patients with a recurrent TBI or significant neurological illness were excluded.	Typically developing children were recruited via social networks of researchers to obtain gender- and age-matched (maximum of 6 months) controls for each TBI patient.
**Cohort**	**Recruitment**	**Inclusion criteria**	**Exclusion criteria**
BCM (all)*	TBI and trauma control participants were recruited from Level 1 trauma centers in Houston, Dallas, and Miami.	1) for TBI group: non-penetrating complicated mild or msTBI (moderate-severe) (post-resuscitation GCS score between 3 and 12); 2) 10-18 years of age; 3) right-handed; 4) normal visual acuity or vision corrected with contact lenses/eyeglasses; and 5) fluent in English or Spanish (the neuropsychological tests used in this study were translated into Spanish and administered by bilingual examiners).	1) history of neurological illness, such as prior msTBI, brain tumor or severe seizures; 2) motor deficits that prevent the subject from being examined; 3) history of psychosis, Tourette's Disorder, learning disability, mental retardation, autism or substance abuse. These conditions are associated with cognitive impairments that might overlap with those caused by TBI. Participants with contraindications to undergoing an MRI scan were excluded.
Murdoch	Children with TBI were enrolled in the study at the time of injury and represented consecutive admissions to a Level 1 trauma center and ED in Melbourne, Australia	(1) documented evidence of accidental TBI, including a period of altered consciousness or presence of at least two postconcussive symptoms; (2) medical records sufficiently detailed to determine injury severity, including the Glasgow Coma Scale (GCS); and (3) child and at least one parent fluent in English.	(1) non-accidental head injuries; (2) parent-reported history of congenital, developmental, or psychiatric condition, including history of ADHD, ASD, and Specific Learning Disorder; (3) previous TBI based on parent-report.

© 2023 Keleher F et al. *JAMA Network Open*

UTHouston	TBI and trauma control participants were recruited from Level 1 trauma center and ED in Houston	Inclusion criteria for TBI, OI, and healthy groups included age at injury between 8 and 15 years, proficiency in English and parent proficient in English or Spanish, residence within 125 miles, no history of prior treated TBI, no preinjury history of major neuropsychiatric disorder, no prior hospitalizations for anxiety or depression, and no history of type 1 or type 2 diabetes. Injuries were sustained in vehicle incidents.	1) prior history of major neuropsychiatric disorder that would complicate assessment of the impact of injury on behavioral outcomes; 2) metabolic disorder; and 3) prior medically attended TBI

© 2023 Keleher F et al. *JAMA Network Open*

eTable 2. Scanner Details

For each cohort the scanner manufacturer and model are listed, along with field strength and voxel size (in mm).

Cohort	Scanner	Field strength (T)	Voxel size (mm)
RAPBI	Siemens TrioTim	3T	1x1x1
Pilot-RAPBI	Siemens Sonata	1.5T	1x1x1
NCH	Siemens Prisma	3T	1x1x1
Kennedy Krieger	Philips	3T	1x1x1
LLU	Siemens TrioTim	3T	1x1x1
Deakin1	Siemens Magnetom Trio	3T	1x1x1
Deakin2	Philips Intera	3T	1x1x1.1
BCM1	Philips Intera or Achieva	1.5T	1x1x1
BCM2	Philips Intera	3T	1x1x1
BCM3	Philips Achieva	3T	1x1x1
Murdoch	Siemens TrioTim	3T	1x0.5x0.5
UTHouston	Philips	3T	1x1x1

eMethods

Outlier Removal

Across segmentation regions, between 7-19 outliers were removed from the 783 scans. Having an outlier volume for one region did not exclude a participant entirely, but rather just from those regional analyses. Participants whose total cerebellum volume was an outlier were removed from all analyses (N = 13). There were no significant differences in the QC fail rate between msTBI and non-TBI for any of the regions

BRIEF

The total number of participants who completed the BRIEF was 421 (msTBI; n = 232, non-TBI; n = 189). The average, SD, and range of *T*-scores for the BRIEF measures within the msTBI group were as follows: for BRI, M = 51.2±11.4; for MCI, M = 52.8±11.3; for GEC, M = 52.4±11.4. Statistical outliers (>3 SDs) were removed (BRI = 7, MCI = 2, GEC = 4).

Additional Cognitive Measures

The Delis-Kaplan Executive Function System (D-KEFS) is a standardized battery of neuropsychological tests used to evaluate higher-order cognitive function in children and adults. For the current study, 231 participants completed the D-KEFS. The sub-test utilized in the current study was the D-KEFS Trail Making Test (TMT) conditions 3 and 4, which involve a visuo-motor sequencing task that measures processing speed (condition 3) and cognitive switching (condition 4). The total number of participants that completed the D-KEFS was 231 (msTBI; n = 110, non-TBI; n = 121). The average, SD and range of standardized scores for the D-KEFS measures were as follows: TMT 3, M = 9.78±9.90, range = 1-15; TMT 4, M = 9.17±5.66, range = 1-15. Higher scores indicate better performance.

© 2023 Keleher F et al. *JAMA Network Open*

eFigure 1. ACAPULCO Segmentation The parcellation of the cerebellum according to ACAPULCO. Images are viewed in radiology space where image left corresponds to the subject's right side. CM=corpus medullare.

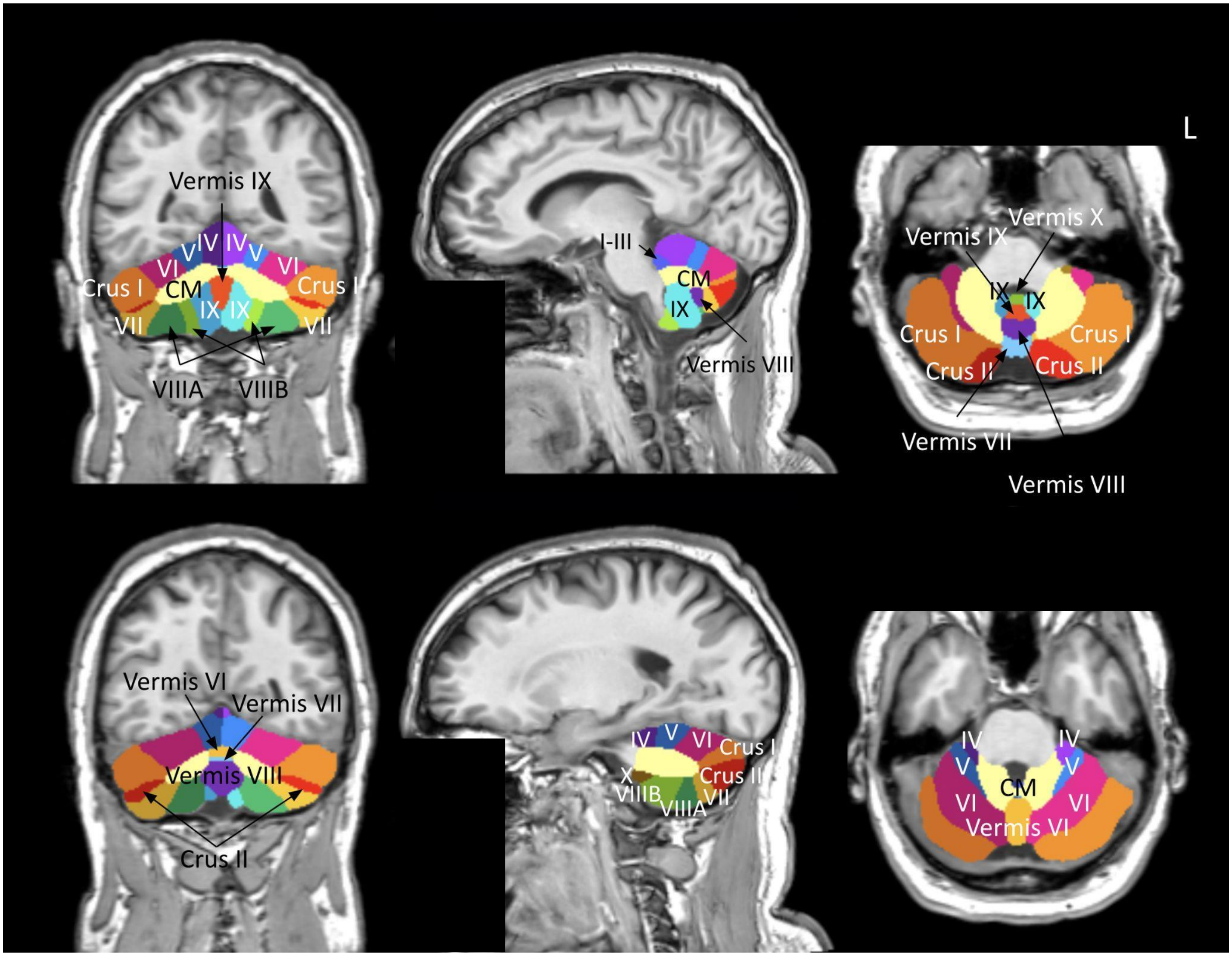

© 2023 Keleher F et al. *JAMA Network Open*

eTable 3. Quality Control Exclusions by ROI and Site

Outlier / visual QC exclusion counts by cerebellar subregion per site.

Region	Subregion	RAPBI	Pilot-RAPBI	NCH	Kennedy-Krieger	LLU	Deakin1	Deakin2	BCM1	BCM2	BCM3	Murdoch	UTHouston	Total
Total Volume		5	3	0	5	4	1	4	18	1	7	1	0	49
Corpus Medullare		6	3	1	7	7	3	1	22	0	8	1	0	59
Anterior Lobe	Lobule I.III	6	3	1	5	4	1	3	20	1	8	1	1	54
	Lobule IV	9	4	1	4	8	1	2	48	1	9	2	1	90
	Lobule V	9	4	1	5	5	2	2	57	2	8	0	1	96
Posterior Lobe	Lobule VI	14	6	9	5	4	2	3	67	1	8	2	0	121
	Crus I	47	22	6	3	14	9	22	105	3	10	24	3	268
	Crus II	20	16	8	7	10	5	9	52	4	8	2	1	142
	Lobule VII	18	8	16	4	7	2	4	45	1	9	2	1	117
	Lobule VIIIA	16	8	11	5	10	3	5	75	1	8	3	8	153
	Lobule VIIIB	11	9	6	5	12	2	4	63	2	8	3	1	126
	Lobule IX	9	5	2	4	7	1	1	52	2	9	2	1	95
Flocculonodular Lobe	Lobule X	6	4	1	4	5	1	1	20	1	8	1	0	52
Vermis	Vermis X	6	3	0	5	6	1	1	21	1	8	1	0	53
	Vermis VI	5	3	1	7	6	1	1	19	1	8	1	0	53
	Vermis VII	8	3	0	4	5	2	1	21	2	9	2	1	58
	Vermis VIII	5	3	1	5	5	1	1	19	1	8	1	0	50
	Vermis IX	6	3	0	6	5	1	1	23	1	8	3	1	58

© 2023 Keleher F et al. *JAMA Network Open*

eTable 3. Quality Control Exclusions by ROI and Site

Outlier / visual QC exclusion counts by cerebellar subregion per site.

Region	Subregion	RAPBI	Pilot-RAPBI	NCH	Kennedy-Krieger	LLU	Deakin1	Deakin2	BCM1	BCM2	BCM3	Murdoch	UTHouston	Total
Total Volume		5	3	0	5	4	1	4	18	1	7	1	0	49
Corpus Medullare		6	3	1	7	7	3	1	22	0	8	1	0	59
	Lobule I.III	6	3	1	5	4	1	3	20	1	8	1	1	54

© 2023 Keleher F et al. *JAMA Network Open*

eTable 4. DTI Protocol Details

Cohort	Voxel size (mm)	Number of gradient directions and b-value (s/mm^2)	No. b0 volumes
RAPBI	2x2x2	64 at b=1000	8
Pilot RAPBI	2.5x2.5x2.5	30 at b=1000	5
NCH	2x2x2	30 at b=700	1
Kennedy Krieger	0.83x0.83x2.2	32 at b=700	1
Loma Linda University	1.2x1.2x3.9	60 at b=1000	2
Deakin-1	2.2x2.2x2.2	64 at b=1000	1
BCM1	2.7x2.7x2.7	15 at b=860	1
BCM2	1.75x1.75x2	32 at b=1000	1
BCM3	1.75x1.75x2	32 at b=1000	1

© 2023 Keleher F et al. *JAMA Network Open*

eAppendix. Li and Ji Modified Bonferroni Correction.

We used a modified Bonferroni correction created by Li and Ji which yielded the effective number of independent variables (V_{eff}) in our analysis as 11 and a significance threshold of $p < 0.05/11 = 0.0045$. A traditional Bonferroni correction was too conservative for our analysis because there are correlations between test statistics (from adjacent subregional cerebellum volumes).

We calculated corrected *p*-values using the following equation:

$$P_{adj} = 1 - (1 - p)^{V_{eff}}$$

Where *p* is the unadjusted *p*-value.

eFigure 2. Flowchart of Analyses

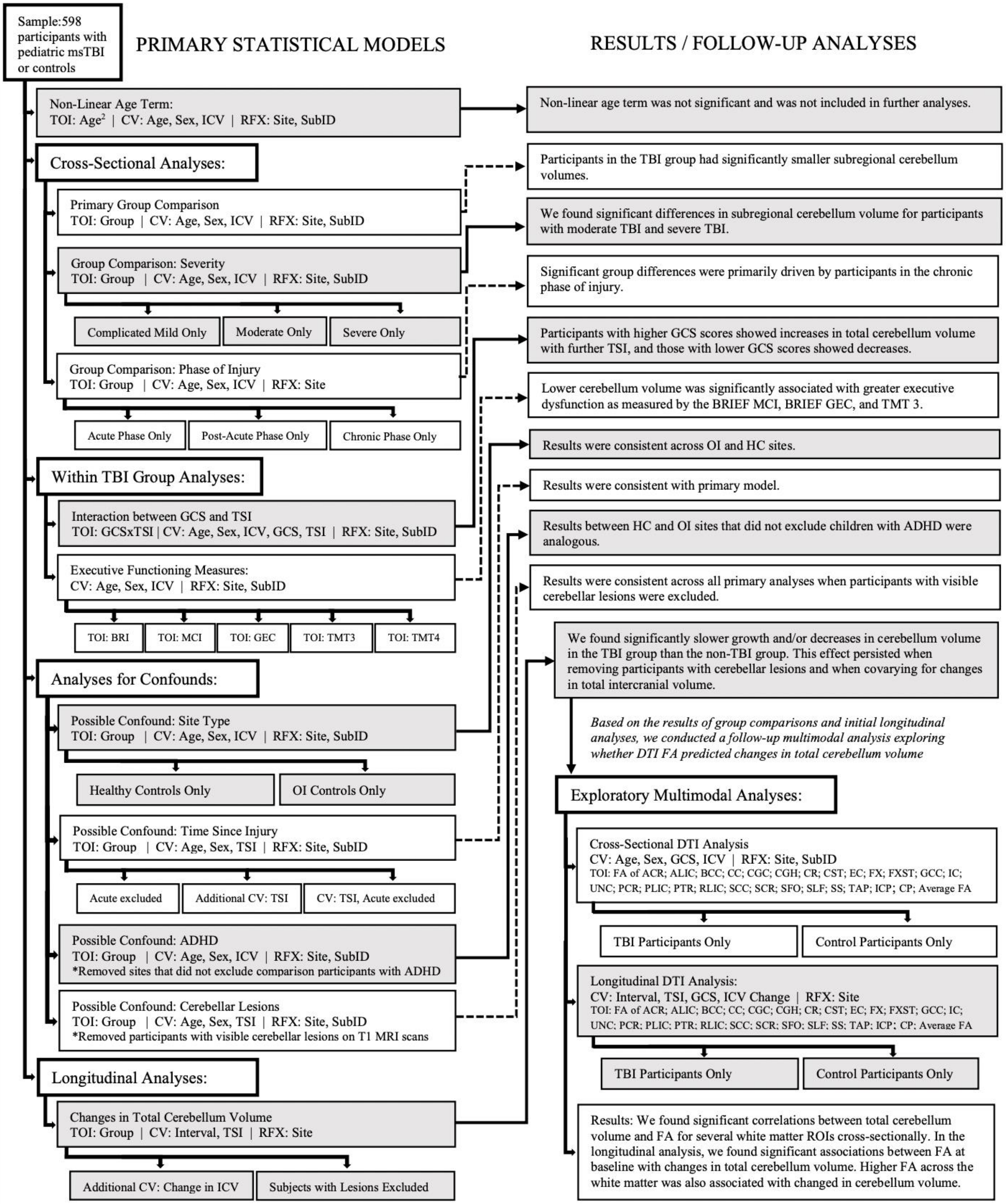

GLOSSARY: **TBI**: Traumatic Brain Injury; **HC**: Healthy Controls; **OI**: Orthopedically Injured Controls; **TOI**: Trait of Interest; **CV**: Covariate; **RFX**: Random Effects; **ICV**: Intercranial Volume; **GCS**: Glasgow Coma Scale score; **TSI**: Time Since Injury; **BRI**: BRIEF Behavioral Regulation Index; **MCI**: BRIEF Metacognition Index; **GEC**: BRIEF Global Executive Composite; **TMT3**: D-KEFS Trail Making Test Condition 3; **TMT4**: D-KEFS Trail Making Test Condition 4; **FA**: Fractional Anisotropy; **ACR:** Anterior Corona Radiata; **ALIC**: Anterior Limb of Internal Capsule; **BCC**: Body of the Corpus Callosum; **CC**: Corpus Callosum; **CGC**: Cingulate Gyrus of Cingulum; **CGH**: Hippocampal Cingulum; **CR**: Corona Radiata; **CST**: Corticospinal Tract ; **EC**: External Capsule; **FX**: Fornix; **FXST**: Fornix/Stria Terminals; **GCC**: Genu of the Corpus Callosum; **IC**: Internal Capsule; **UNC**: Uncinate Fasciculus; **PCR**: Posterior Corona Radiata; **PLIC**: Posterior Limb of Internal Capsule; **PTR**: Posterior Thalamic Radiation; **RLIC**: Retrorenticular Part of Internal Capsule; **SCC**: Splenium; **SCR**: Superior Corona Radiata; **SFO**: Superior Fronto-Occipital Fasciculus; **SLF**: Superior Longitudinal Fasciculus; **SS**: Sagittal Stratum; **TAP**: Tapetum; **ICP**: Inferior Cerebellar Peduncle; **CP**: Cerebellar Peduncle

© 2023 Keleher F et al. *JAMA Network Open*

eTable 5. Group Comparisons in Different Phases of Injury

The p-values, Cohen d-values, and the 95% confidence intervals for d-values are shown for results sorted by phase of injury. Acute<6 weeks post-injury, Post-acute=6 weeks to 6 months since injury, Chronic=>6 months post-injury.

		Acute			Post-Acute			Chronic		
Region	Subregion	*p*-value	Cohen's D	CI	*p*-value	Cohen's D	CI	*p*-value	Cohen's D	CI
Total Volume		0.12	-0.28	[-0.63 to 0.07]	0.06	-0.25	[-0.50 to 0.00]	<0.001	-0.55	[-0.75 to-0.35]
Corpus Medullare		0.03	-0.39	[-0.75 to -0.04]	0.07	-0.23	[-0.48 to 0.02]	<0.001	-0.53	[-0.73 to -0.32]
Anterior Lobe	Lobule I.III	0.62	0.09	[-0.26 to 0.44]	0.57	0.07	[-0.18 to 0.33]	0.02	-0.23	[-0.43 to -0.03]
	Lobule IV	0.49	0.12	[-0.2 to ,0.47]	0.84	-0.03	[-0.28 to 0.23]	0.49	0.07	[-0.13 to 0.27]
	Lobule V	0.98	-0.01	[-0.36 to 0.35]	0.84	-0.03	[-0.28 to 0.23]	0.004	-0.30	[-0.50 to -0.10]
Posterior Lobe	Lobule VI	0.16	-0.25	[-0.60 to 0.10]	0.64	-0.07	[-0.35 to 0.22]	0.03	-0.24	[-0.45 to -0.03]
	Crus I	0.49	0.13	[-0.24 to 0.49]	0.04	-0.33	[-0.64 to -0.01]	0.08	-0.21	[-0.44 to 0.02]
	Crus II	0.10	-0.31	[-0.68 to 0.06]	0.10	-0.24	[-0.51 to 0.04]	<0.001	-0.40	[-0.62 to -0.19]
	Lobule VIIB	0.13	-0.28	[-0.64 to 0.08]	0.61	-0.07	[-0.33 to 0.20]	0.02	-0.26	[-0.46 to -0.05]
	Lobule VIIIA	0.75	-0.06	[-0.42 to 0.30]	0.54	0.09	[-0.19 to 0.36]	0.02	-0.26	[-0.48 to -0.03]
	Lobule VIIIB	0.54	-0.12	[-0.48 to 0.25]	0.02	-0.35	[-0.65 to -0.06]	<0.001	-0.55	[-0.77 to -0.32]
	Lobule IX	0.82	0.04	[-0.32 to 0.40]	0.11	-0.23	[-0.50 to 0.05]	0.05	-0.21	[-0.41 to 0.00]
Flocculonodular Lobe	Lobule X	0.65	0.08	[-0.27 to 0.43]	0.87	0.02	[-0.23 to 0.27]	0.62	0.05	[-0.15 to 0.25]
Vermis	Vermis X	0.94	-0.01	[-0.37 to 0.34]	0.08	-0.23	[-0.48 to 0.03]	0.02	-0.24	[-0.44 to -0.05]
	Vermis VI	0.26	-0.21	[-0.57 to 0.15]	0.47	0.09	[-0.16 to 0.35]	0.28	0.11	[-0.09 to 0.31]
	Vermis VII	<0.001	-0.69	[-1.05 to -0.32]	0.14	-0.19	[-0.45 to 0.06]	0.001	-0.32	[-0.52 to -0.12]
	Vermis VIII	0.80	0.04	[-0.31 to 0.40]	0.06	-0.24	[-0.49 to 0.01]	0.23	-0.12	[-0.32 to 0.08]
	Vermis IX	0.51	-0.12	[-0.48 to 0.24]	0.06	-0.24	[-0.50 to 0.01]	0.001	-0.24	[-0.50 to 0.01]

© 2023 Keleher F et al. *JAMA Network Open*

eTable 6. Group Comparisons With Different TBI Severity Groups

Effects of injury severity as rated by the GCS on cerebellum volumes.The p-values, Cohen d-values, and the 95% confidence intervals for d-values are shown for results sorted by GCS severity. Complicated Mild=13-15, Moderate=9=12, Severe=3-8

		Complicated Mild			Moderate			Severe		
Region	Subregion	*p*-value	Cohen's D	CI	*p*-value	Cohen's D	CI	*p*-value	Cohen's D	CI
Total Volume		0.48	-0.07	[-0.26 to 0.12]	0.01	-0.27	[-0.47 to -0.07]	<0.001	-0.33	[-0.51 to -0.15]
Corpus Medullare		0.09	-0.17	[-0.36 to 0.03]	0.003	-0.30	[-0.50 to -0.10]	<0.001	-0.42	[-0.60 to -0.24]
Anterior Lobe	Lobule I.III	0.91	-0.01	[-0.20 to 0.18]	0.30	-0.11	[-0.31 to 0.09]	0.78	-0.03	[-0.20 to 0.15]
	Lobule IV	0.86	0.02	[-0.18 to 0.21]	0.73	0.04	[-0.17 to 0.24]	0.80	-0.02	[-0.20 to 0.16]
	Lobule V	0.91	0.01	[-0.18 to 0.20]	0.08	-0.18	[-0.38 to 0.02]	0.004	-0.26	[-0.44 to -0.08]
Posterior Lobe	Lobule VI	0.42	0.09	[-0.12 to 0.29]	0.57	-0.06	[-0.28 to 0.15]	0.006	-0.27	[-0.46 to -0.08]
	Crus I	0.93	0.01	[-0.22 to 0.23]	0.83	-0.03	[-0.26 to 0.21]	0.003	-0.33	[-0.54 to -0.11]
	Crus II	0.13	-0.16	[-0.37 to 0.05]	0.01	-0.28	[-0.49 to -0.06]	0.006	-0.28	[-0.47 to -0.08]
	Lobule VIIB	0.19	-0.13	[-0.33 to 0.07]	<0.001	-0.38	[-0.59 to -0.17]	0.02	-0.23	[-0.42 to -0.04]
	Lobule VIIIA	0.77	-0.03	[-0.24 to 0.18]	0.30	-0.12	[-0.34 to 0.10]	0.97	0.00	[-0.19 to 0.20]
	Lobule VIIIB	0.02	-0.26	[-0.47 to -0.04]	0.32	-0.12	[-0.34 to 0.11]	<0.001	-0.38	[-0.58 to -0.18]
	Lobule IX	0.12	-0.16	[-0.37 to 0.04]	0.56	-0.06	[-0.28 to 0.15]	0.01	-0.25	[-0.44 to -0.06]
Flocculonodular Lobe	Lobule X	0.44	-0.08	[-0.27 to 0.12]	0.64	-0.05	[-0.25 to 0.15]	0.52	-0.06	[-0.24 to 0.12]
Vermis	Vermis X	0.22	-0.12	[-0.31 to 0.07]	0.24	-0.12	[-0.32 to 0.08]	0.01	-0.23	[-0.41 to -0.05]
	Vermis VI	0.36	0.09	[-0.10 to 0.29]	0.26	0.12	[-0.09 to 0.32]	0.54	0.06	[-0.12 to 0.24]
	Vermis VII	0.11	-0.16	[-0.35 to 0.04]	0.04	-0.21	[-0.41 to -0.01]	0.06	-0.17	[-0.35 to 0.01]

© 2023 Keleher F et al. *JAMA Network Open*

Vermis VIII	0.19	-0.13	[-0.32 to 0.06]	0.44	-0.08	[-0.28 to 0.12]	0.09	-0.16	[-0.34 to 0.02]
Vermis IX	0.76	-0.03	[-0.22 to 0.16]	0.18	-0.14	[-0.34 to 0.06]	0.03	-0.19	[-0.37 to -0.01]

The p-values, Cohen d-values, and the 95% confidence intervals for d-values are shown for results sorted by GCS severity. Complicated Mild=13-15, Moderate=9=12, Severe=3-8

© 2023 Keleher F et al. *JAMA Network Open*

eFigure 3. Group Differences in Longitudinal Changes in Cerebellum Volume

Longitudinal changes in total cerebellum volume are shown for the subset of participants with high-quality longitudinal data, outliers (3SD) removed (N=72). The boxplot shows the percent volume change (normalized residuals) for the non-TBI group (pink, N=34), and the msTBI group (blue, N=38).

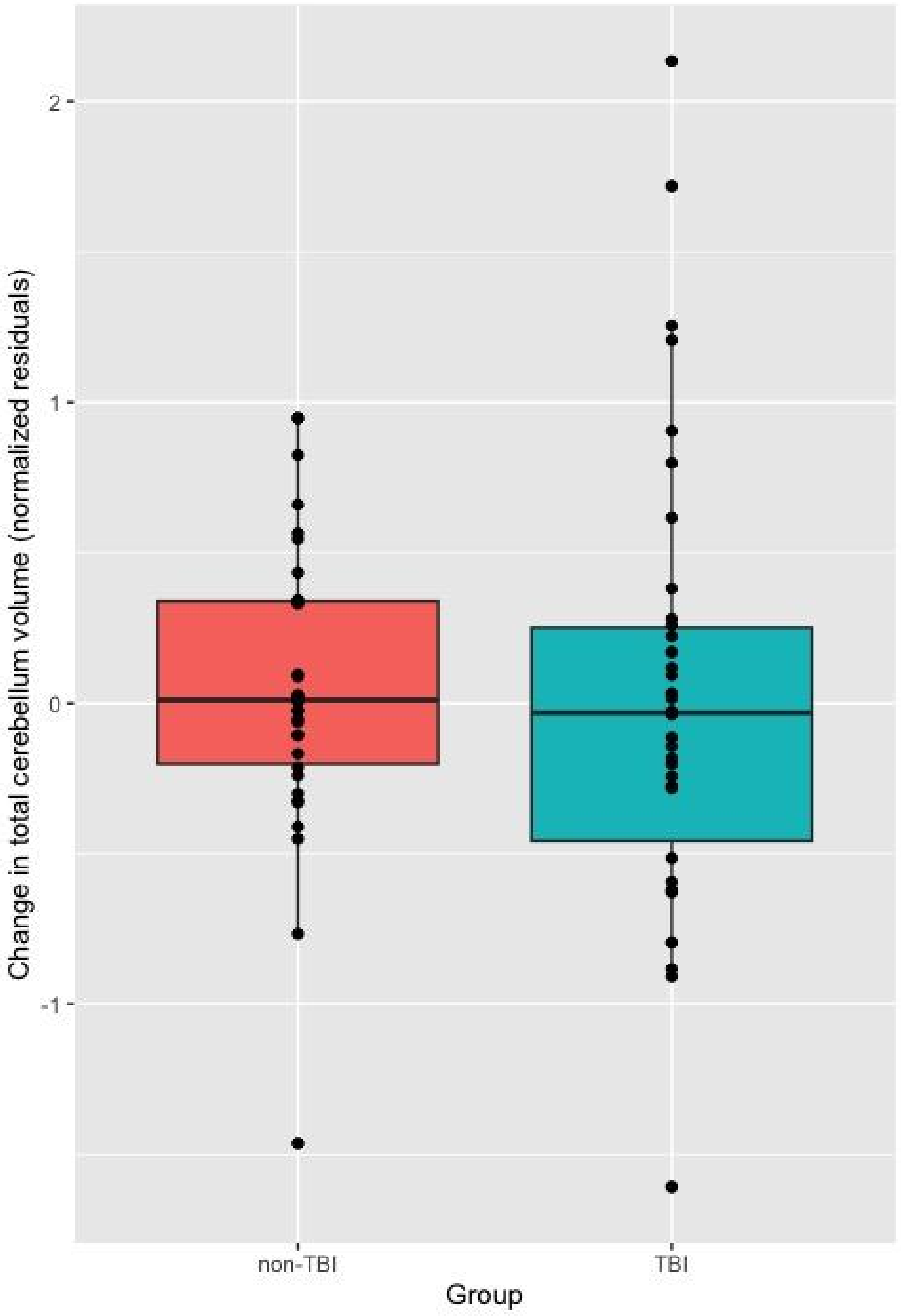

© 2023 Keleher F et al. *JAMA Network Open*

eFigure 4. Association Between Age at Injury and Percent Change in Total Cerebellum Volume for the Subset of TBI Participants With Longitudinal Data Available With Outliers Winsorized to 3SD

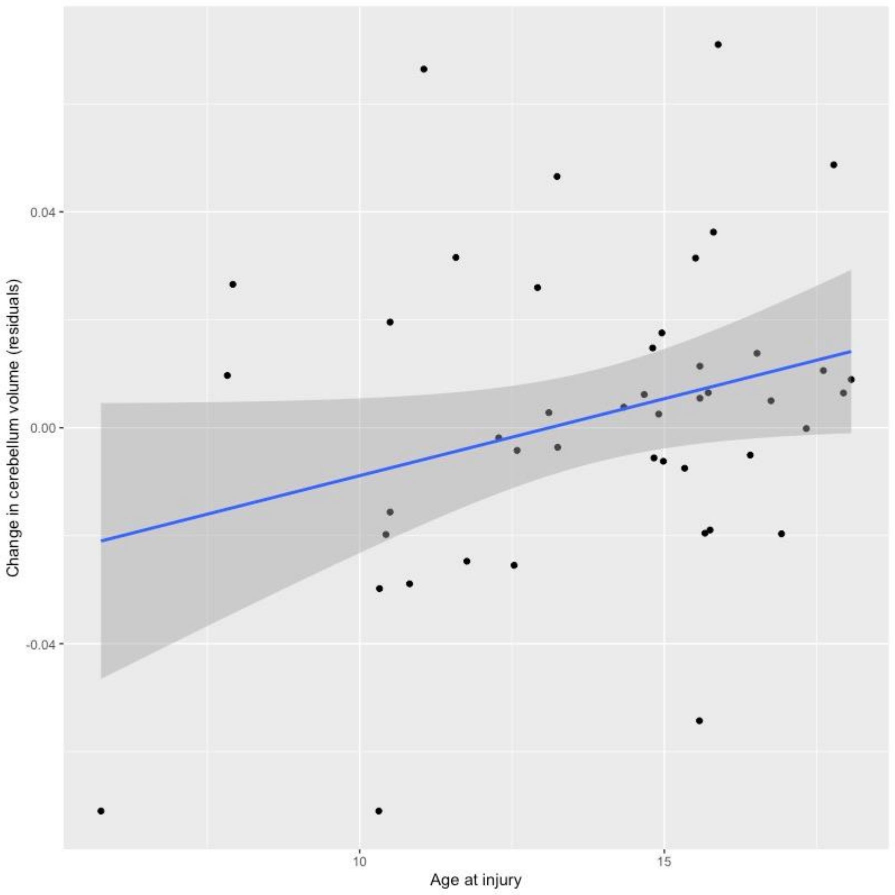

© 2023 Keleher F et al. *JAMA Network Open*

eResults

Non-linear Age Term

We first tested whether a nonlinear age term (age^2) should be included in statistical models along with age, sex, and ICV. Age^2 was not significant (p's>0.10) , so it was not included in subsequent analyses.

Secondary Group Comparisons

We tested several additional models covarying for TSI, excluding participants in the acute phase (< 7 weeks post-injury), or both excluding acute and covarying for TSI. Results were consistent with our primary model (**eTable 7**). Further, we examined group differences separately for sites that had HC versus those with OI controls (**eTable 8**). We found significant differences only with the HC comparison, while differences between msTBI and OI were no longer significant. However, ADHD is a common pre-/comorbidity of TBI[38] and is associated with cerebellar changes,[31] so we also examined only HC and OI sites that excluded children with ADHD. When excluding HC and OI cohorts that did not exclude children with ADHD, the HC and OI results were comparable (**eTable 8**).

Exploratory Multimodal MRI Analyses

We found that total cerebellum volume change was associated with higher FA in the body of the corpus callosum, cingulum, hippocampal cingulum, corona radiata, fornix, inferior cerebellar peduncle, posterior thalamic radiation, retrolenticular limb of internal capsule, superior longitudinal fasciculus, sagittal stratum, and tapetum associated with increases in cerebellum volume in the msTBI group (**eTable 9** and **eFigure 5**). Higher average FA across the white matter was also associated with changes in cerebellum volume.

Interactions

We did not find any significant interactions between group and age or group and sex. There were also no significant interactions within the msTBI group between age at injury and TSI or between age at injury and GCS when covarying for age at scan (**eTable 10**). We found a significant interaction between TSI and GCS for total cerebellum volume ($b = 6.3$, $p < .001$; **eFigure 6**), with participants with higher GCS scores (milder injuries) showing volume increases with further time since injury and those with lower GCS scores showing decreases in volume over time.

D-KEFS Trail Making Test

Lower cerebellar volume was also associated with slower processing speed in participants with msTBI. For the D-KEFS TMT, there was a significant association between the condition 3 score and the volume of Lobule I-III ($b = 4.0$, $p = 0.022$). Results are summarized in **eTable 11.**

© 2023 Keleher F et al. *JAMA Network Open*

eTable 7. **Supplemental Group Comparisons**

Results are shown for the main group comparison 1) excluding participants within 7 weeks of injury, 2) excluding participants within 7 weeks of injury and covarying for time since injury (TSI), and 3) covarying for TSI. The p-values, Cohen d-values, and the 95% confidence intervals for d-values are shown for each model

		Post-Acute / Chronic Only			Post Acute / Chronic Only + covaried for TSI			All Phases + covaried for TSI		
Region	Subregion	*p*-value	Cohen's D	CI	*p*-value	Cohen's D	CI	*p*-value	Cohen's D	CI
Total Volume		<0.001	-0.46	[-0.62 to -0.29]	<0.001	-0.37	[-0.55 to -0.19]	<0.001	-0.37	[-0.53 to -0.21]
Corpus Medullare		<0.001	-0.38	[-0.55 to -0.21]	0.001	-0.30	[-0.47 to -0.12]	<0.001	-0.28	[-0.44 to -0.12]
Anterior Lobe	Lobule I.III	0.11	-0.14	[-0.30 to 0.03]	0.47	-0.07	[-0.24 to 0.11]	0.74	-0.03	[-0.19 to 0.13]
	Lobule IV	0.73	0.03	[-0.14 to 0.20]	0.75	0.03	[-0.15 to 0.21]	1.0	0.00	[-0.16 to 0.16]
	Lobule V	0.01	-0.21	[-0.38 to -0.05]	0.15	-0.13	[-0.31 to 0.05]	0.14	-0.12	[-0.28 to 0.04]
Posterior Lobe	Lobule VI	0.05	-0.18	[-0.36 to 0.00]	0.35	-0.09	[-0.29 to 0.10]	0.03	-0.19	[-0.36 to -0.02]
	Crus I	0.005	-0.30	[-0.50 to -0.09]	0.01	-0.3	[-0.51 to -0.08]	0.01	-0.25	[-0.44 to -0.06]
	Crus II	<0.001	-0.36	[-0.54 to -0.18]	0.005	-0.28	[-0.47 to -0.08]	0.005	-0.25	[-0.42 to -0.08]
	Lobule VIIB	0.002	-0.28	[-0.46 to -0.10]	0.003	-0.28	[-0.47 to -0.09]	0.003	-0.25	[-0.42 to -0.09]
	Lobule VIIIA	0.60	-0.05	[-0.24 to 0.14]	0.57	-0.06	[-0.28 to 0.15]	0.64	-0.04	[-0.22 to 0.14]
	Lobule VIIIB	<0.001	-0.43	[-0.62 to -0.24]	<0.001	-0.38	[-0.59 to -0.18]	<0.001	-0.35	[-0.54 to -0.17]
	Lobule IX	0.06	-0.17	[-0.35 to 0.01]	0.04	-0.20	[-0.39 to -0.01]	0.06	-0.16	[-0.33 to 0.01]
Flocculonodular Lobe	Lobule X	0.99	0.00	[-0.17 to 0.17]	0.69	-0.04	[-0.21 to 0.14]	0.89	-0.01	[-0.17 to 0.15]
Vermis	Vermis X	0.002	-0.27	[-0.44 to -0.10]	<0.001	-0.32	[-0.50 to -0.14]	0.001	-0.27	[-0.43 to -0.11]
	Vermis VI	0.11	0.13	[-0.03 to 0.30]	0.14	0.13	[-0.04 to 0.31]	0.58	0.05	[-0.12 to 0.21]
	Vermis VII	0.08	-0.15	[-0.31 to 0.02]	0.09	-0.15	[-0.33 to 0.02]	0.005	-0.23	[-0.40 to -0.07]
	Vermis VIII	0.10	-0.14	[-0.30 to 0.03]	0.02	-0.22	[-0.39 to -0.04]	0.02	-0.19	[-0.35 to -0.03]

© 2023 Keleher F et al. *JAMA Network Open*

eTable 7. **Supplemental Group Comparisons**

Results are shown for the main group comparison 1) excluding participants within 7 weeks of injury, 2) excluding participants within 7 weeks of injury and covarying for time since injury (TSI), and 3) covarying for TSI. The p-values, Cohen d-values, and the 95% confidence intervals for d-values are shown for each model

		Post-Acute / Chronic Only			Post Acute / Chronic Only + covaried for TSI			All Phases + covaried for TSI		
Region	Subregion	*p*-value	Cohen's D	CI	*p*-value	Cohen's D	CI	*p*-value	Cohen's D	CI
Total Volume		<0.001	-0.46	[-0.62 to -0.29]	<0.001	-0.37	[-0.55 to -0.19]	<0.001	-0.37	[-0.53 to -0.21]
Corpus Medullare		<0.001	-0.38	[-0.55 to -0.21]	0.001	-0.30	[-0.47 to -0.12]	<0.001	-0.28	[-0.44 to -0.12]
	Lobule I.III	0.11	-0.14	[-0.30 to 0.03]	0.47	-0.07	[-0.24 to 0.11]	0.74	-0.03	[-0.19 to 0.13]
	Vermis IX	0.01	-0.21	[-0.38 to -0.05]	0.06	-0.17	[-0.35 to 0.01]	0.02	-0.20	[-0.36 to -0.04]

© 2023 Keleher F et al. *JAMA Network Open*

eTable 8. **Group Comparisons With Different Control Groups**

Effects of site type (OI comparisons or healthy controls), and presence of ADHD in cohort on cerebellum volumes.The p-values, Cohen d-values, and the 95% confidence intervals for d-values are shown for results sorted by site type.

		Healthy Controls			OI Comparisons			Healthy Controls (no ADHD)			OI Comparisons (no ADHD)		
Region	Subregion	*p*-value	Cohen's D	CI	*p*-value	Cohen's D	CI	*p*-value	Cohen's D	CI	*p*-value	Cohen's D	CI
Total Volume		<0.001	-0.42	[-0.60 to -0.24]	0.02	-0.13	[-0.38 to 0.12]	<0.001	-0.64	[-0.88 to -0.39]	0.01	-0.38	[-0.69 to -0.08]
Corpus Medullare		<0.001	-0.33	[-0.51 to -0.15]	0.04	-0.13	[-0.38 to 0.11]	<0.001	-0.67	[-0.92 to -0.42]	0.02	-0.38	[-0.68 to -0.07]
Anterior Lobe	Lobule I.III	0.27	-0.03	[-0.20 to 0.15]	0.81	-0.05	[-0.36 to 0.26]	0.44	-0.10	[-0.34 to 0.15]	0.43	-0.12	[-0.43 to 0.18]
	Lobule IV	0.59	-0.02	[-0.20 to 0.16]	0.34	-0.11	[-0.39 to 0.16]	0.48	-0.09	[-0.33 to 0.16]	0.22	0.20	[-0.12 to 0.51]
	Lobule V	0.04	-0.26	[-0.44 to -0.08]	0.20	-0.07	[-0.32 to 0.17]	0.04	-0.26	[-0.50 to -0.02]	0.27	-0.17	[-0.48 to 0.14]
Posterior Lobe	Lobule VI	0.004	-0.27	[-0.46 to -0.08]	0.95	-0.16	[-0.41 to 0.09]	0.01	-0.33	[-0.58 to -0.08]	0.66	0.09	[-0.31 to 0.49]
	Crus I	0.008	-0.28	[-0.49 to -0.08]	0.75	-0.05	[-0.36 to 0.26]	0.004	-0.45	[-0.75 to -0.14]	0.22	-0.29	[-0.75 to 0.17]
	Crus II	<0.001	-0.28	[-0.47 to -0.08]	0.41	0.12	[-0.13 to 0.37]	<0.001	-0.65	[-0.93 to -0.37]	0.16	-0.25	[-0.59 to 0.09]
	Lobule VIIB	0.01	-0.23	[-0.42 to -0.04]	0.02	0.01	[-0.28 to 0.30]	0.004	-0.38	[-0.64 to -0.12]	0.03	-0.36	[-0.67 to -0.04]
	Lobule VIIIA	0.08	0.00	[-0.19 to 0.20]	0.48	-0.33	[-0.59 to -0.06]	0.05	-0.26	[-0.52 to 0.00]	0.68	0.07	[-0.27 to 0.42]
	Lobule VIIIB	<0.001	-0.38	[-0.58 to -0.18]	0.03	0.11	[-0.19 to 0.41]	<0.001	-0.53	[-0.79 to -0.26]	0.16	-0.29	[-0.70 to 0.11]
	Lobule IX	0.31	-0.25	[-0.44 to -0.06]	0.05	-0.35	[-0.66 to -0.04]	0.38	-0.11	[-0.36 to 0.14]	0.04	-0.40	[-0.78 to -0.01]
Flocculonodular Lobe	Lobule X	0.50	-0.06	[-0.24 to 0.12]	0.56	-0.29	[-0.57 to -0.01]	0.20	0.16	[-0.08 to 0.40]	0.62	-0.08	[-0.38 to 0.23]
	Vermis X	0.06	-0.23	[-0.41 to -0.05]	0.10	-0.26	[-0.51 to -0.02]	0.04	-0.26	[-0.50 to -0.02]	0.28	-0.17	[-0.48 to 0.14]
	Vermis VI	0.62	0.06	[-0.12 to 0.24]	0.14	-0.30	[-0.54 to -0.05]	0.95	-0.01	[-0.25 to 0.23]	0.12	0.25	[-0.06 to 0.56]

Vermis	Vermis VII	0.03	-0.17	[-0.35 to 0.01]	0.09	-0.21	[-0.46 to 0.04]	0.49	-0.09	[-0.33 to 0.16]	0.18	-0.21	[-0.52 to 0.10]
	Vermis VIII	0.07	-0.16	[-0.34 to 0.02]	0.29	0.19	[-0.06 to 0.44]	0.03	-0.27	[-0.52 to -0.03]	0.18	-0.21	[-0.52 to 0.10]
	Vermis IX	0.002	-0.19	[-0.37 to -0.01]	0.31	-0.22	[-0.47 to 0.03]	0.02	-0.30	[-0.55 to -0.06]	0.89	0.02	[-0.29 to 0.33]

© 2023 Keleher F et al. *JAMA Network Open*

eTable 9. **Interactions**

Interaction effects between relevant independent variables (group and age, group and sex, age at injury and time since injury, age at injury and GCS score, and time since injury and GCS score).The p-values and beta-values are shown for interaction models.

		Group x Age		Group x Sex		AgeatInj x TSI		AgeatInj x GCS		TSI x GCS	
Region	Subregion	*p*-value	*b*-value	*p*-value	*b*-value	*p*-value	*b*-value	*p*-value	*b*-value	*p*-value	*b*-value
Total Volume		0.69	136.03	0.36	-1952.13	0.34	-0.05	0.07	-24.53	<0.001	6.33
Corpus Medullare		0.37	-72.12	0.33	-496.09	0.76	0.06	0.79	2.20	0.05	0.76
Anterior Lobe	Lobule I.III	0.30	6.33	0.06	-71.05	0.71	0.02	0.76	-0.11	0.52	-0.02
	Lobule IV	0.85	-2.82	0.90	12.30	0.48	-0.01	0.83	0.31	0.13	0.11
	Lobule V	0.13	19.18	0.55	46.60	0.37	-0.04	0.86	-0.47	0.22	0.09
Posterior Lobe	Lobule VI	0.45	27.70	0.40	-187.92	0.07	0.15	0.30	-6.54	0.01	0.42
	Crus I	0.39	-56.90	0.60	-214.70	0.81	-0.05	0.01	-24.50	0.003	1.0
	Crus II	0.94	-3.13	0.46	189.56	0.65	0.22	0.74	-5.28	0.93	0.02
	Lobule VIIB	0.14	48.69	0.83	43.63	0.25	-0.12	0.25	0.39	0.01	0.52
	Lobule VIIIA	0.20	-43.13	0.75	-65.60	0.31	-0.07	0.94	1.40	0.12	0.34
	Lobule VIIIB	0.94	-1.42	0.45	-89.77	0.36	0.05	0.65	-1.37	0.04	0.25
	Lobule IX	0.81	4.76	0.45	-91.81	0.43	0.08	0.65	-3.37	0.05	0.17
Flocculonodular Lobe	Lobule X	0.13	4.36	0.14	-26.03	0.04	1.11	0.80	-100.16	0.41	0.01
Vermis	Vermis X	0.23	2.18	0.61	-5.85	0.57	0.00	0.97	0.01	0.92	0.00
	Vermis VI	0.13	10.86	0.10	-72.99	0.47	0.02	0.91	0.13	0.32	0.04
	Vermis VII	0.12	7.87	0.66	13.96	0.95	0.00	0.68	0.30	0.20	0.03

© 2023 Keleher F et al. *JAMA Network Open*

Vermis VIII	0.81	-2.16	0.06	-104.43	0.78	-0.01	0.03	-3.25	0.27	0.04
Vermis IX	0.02	10.60	0.53	17.17	0.11	0.03	0.14	-1.02	0.37	0.02

© 2023 Keleher F et al. *JAMA Network Open*

eFigure 5. Interaction Between Time Since Injury and Injury Severity
The interaction between time since injury in weeks and Glasgow Coma Scale is shown for total cerebellum volume (normalized residuals accounting for age, sex, ICV, and random effects of site and subject). TSI is truncated at 4 years. Light blue dots and dotted line are for participants with GCS in the bottom tertile (most severe), dark blue dots and solid line are for participants with GCS in the top tertile (least severe), with the middle blue dots and dashed line for participants in the middle tertile.

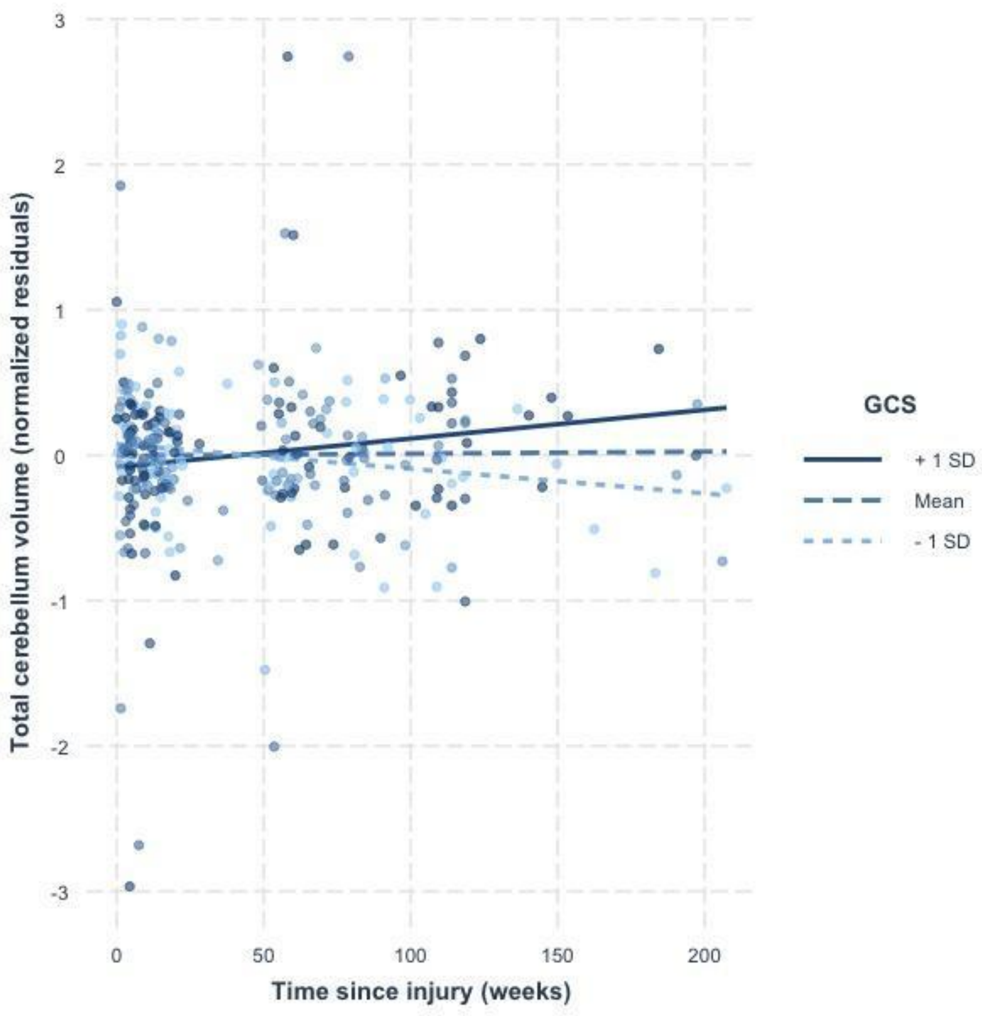

© 2023 Keleher F et al. *JAMA Network Open*

eTable 10. Cross-Sectional and Longitudinal Associations Between FA and Total Cerebellum Volume in the TBI Group

Longitudinal associations are between FA at first scan and changes in total cerebellum volume. FA=fractional anisotropy, ACR=anterior corona radiata, ALIC=anterior limb of internal capsule, BCC=body of corpus callosum, CC=corpus callosum, CGC=cingulum, CGH=hippocampal cingulum, CP=cerebellar peduncle, CR=corona radiata, CST=corticospinal tract, EC=external capsule, FX=fornix, FXST=fornix stria terminalis, GCC=genu of corpus callosum, IC=internal capsule, ICP=inferior cerebellar peduncle, PCR=posterior corona radiata, PLIC=posterior limb of internal capsule, PTR=posterior thalamic radiation, RLIC=retrolenticular limb of internal capsule, SCC=splenium of corpus callosum, SCP=superior cerebellar peduncle, SCR=superior corona radiata, SFO=superior fronto-occipital fasciculus, SLF=superior longitudinal fasciculus, SS=sagittal stratum, TAP=tapetum, UNC=uncinate. Significant associations are **bolded**.

	Cross-sectional, covarying for GCS (N=252)		Longitudinal, covarying for interval, TSI, and GCS (N=32)	
ROI	*t-value*	*p-value*	*t-value*	*p-value*
AverageFA	4.1	<0.001	3.2	0.005
ACR	1.6	0.12	2.3	0.03
ALIC	1.9	0.06	2.0	0.06
BCC	3.4	0.002	3.1	0.006
CC	3.6	<0.001	2.9	0.008
CGC	3.6	<0.001	3.1	0.005
CGH	1.9	0.07	2.3	0.03
CP	2.7	0.01	0.3	0.76
CR	2.3	0.03	2.3	0.03
CST	2.9	0.006	0.9	0.36
EC	2.8	0.008	1.6	0.13
FX	3.5	0.001	3.8	0.001
FXST	2.9	0.005	1.2	0.23
GCC	1.4	0.16	1.1	0.29
IC	2.2	0.03	1.8	0.10
ICP	2.6	0.01	2.5	0.02
PCR	1.9	0.06	2.3	0.03
PLIC	1.4	0.16	1.0	0.32
PTR	2.4	0.02	2.4	0.03
RLIC	2.8	0.007	3.2	0.005
SCC	3.8	<0.001	2.3	0.03
SCP	1.8	0.08	0.7	0.48
SCR	2.5	0.02	1.9	0.08
SFO	1.2	0.24	2.0	0.05
SLF	3.8	<0.001	2.9	0.009
SS	2.5	0.02	2.4	0.03
TAP	3.4	0.002	3.3	0.004
UNC	2.8	0.007	0.0	0.98

© 2023 Keleher F et al. *JAMA Network Open*

eFigure 6. White Matter Regions Associated With Longitudinal Changes in Total Cerebellum Volume in the TBI Group
Color corresponds to the Pearson's *r* as depicted in the color bar.

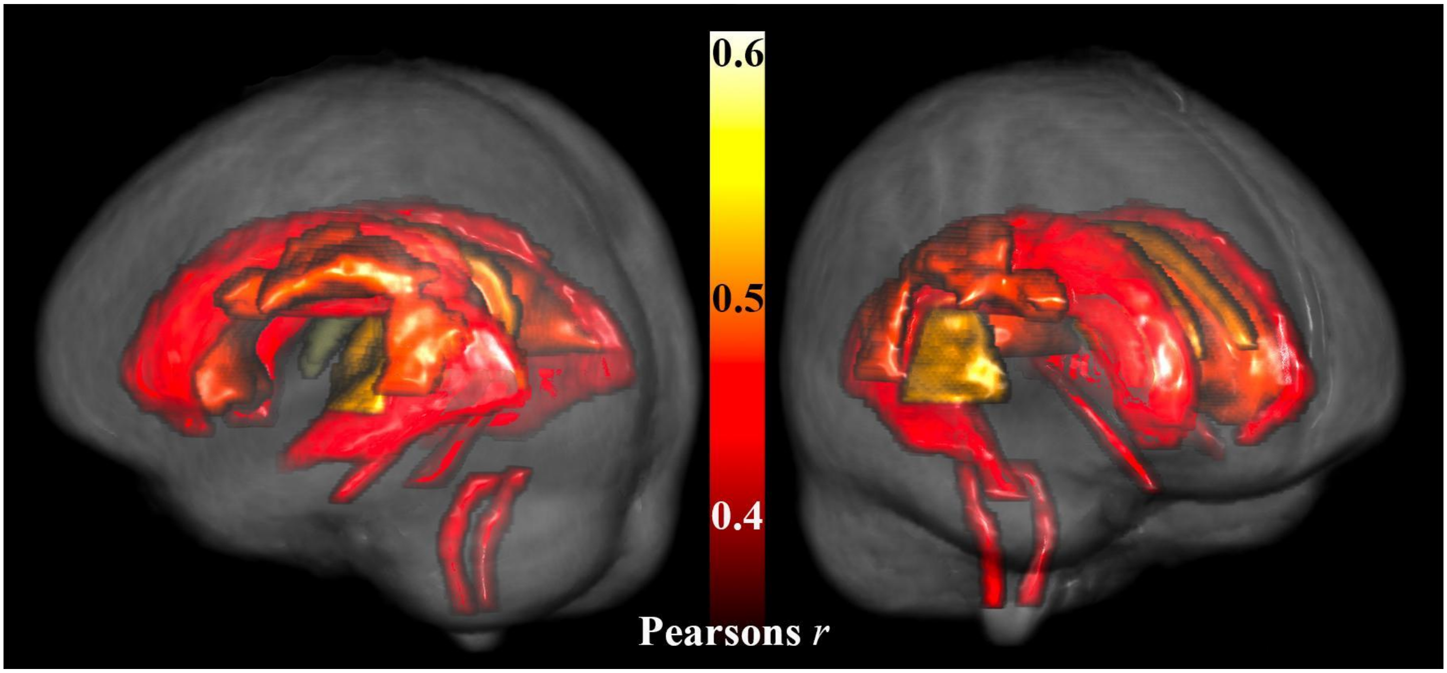

© 2023 Keleher F et al. *JAMA Network Open*
