# Supplementary material for: Multimodal Analysis of Secondary Cerebellar Alterations After Pediatric Traumatic Brain Injury
Source: JAMA Netw Open. 2023 Nov 15;6(11):e2343410. doi: 10.1001/jamanetworkopen.2023.43410 (PMC10652147; doi:10.1001/jamanetworkopen.2023.43410)
Supplement: Supplement 2. — Data Sharing Statement [file jamanetwopen-e2343410-s002.pdf]

Data Sharing Statement

Data

Data available: No

Additional Information

Explanation for why data not available: Some of our data come from international groups and thus require a data transfer agreement to share. Researchers are welcome to join our group and submit a proposal and sites can opt in to sharing data.
